# An AI-Ready Phosphorylation Meta-Analysis for *Saccharomyces cerevisiae*

**DOI:** 10.1021/acs.jproteome.5c01263

**Published:** 2026-05-04

**Authors:** Ellen L Boswell, Kerry A Ramsbottom, Jun Fan, Yasset Perez-Riverol, Emily H Bowler-Barnett, Zhi Sun, Shahram Mesdaghi, Daniel J Rigden, Maria J Martin, Eric W Deutsch, Juan Antonio Vizcaíno, Andrew R Jones

**Affiliations:** † Institute of Systems, Molecular and Integrative Biology, 4591 University of Liverpool, Liverpool L69 7BE, U.K.; ‡ European Molecular Biology Laboratory, 9470 EMBL-European Bioinformatics Institute (EMBL-EBI), Hinxton, Cambridge CB10 1SD, U.K.; § 7268 Institute for Systems Biology, Seattle, Washington 98109, United States

**Keywords:** phosphoproteomics, *Saccharomyces cerevisiae*, false localization rate, AI-ready data

## Abstract

We, the PTMeXchange Consortium, present a meta-analysis of eight high-quality data sets to map 56,694 phosphosites in brewer’s yeast (*Saccharomyces cerevisiae*) using strict control for false identifications. Each site has been classified into the Gold-Silver-Bronze confidence categories. First, we identified 55 significant motifs and grouped these into kinase classes to perform pathway enrichment analysis. Next, we leveraged disorder region predictions and AlphaFold 3′s ability to consider post-translational modifications (PTMs) when modeling proteins to understand the structural context of phosphosites. Here, we determined that phosphorylation tends to occur on disordered serine and threonine residues. AlphaFold predictions suggest that phosphosites induce alpha helices to form in proteins, although many “induced helices” appear to be unusually short and require further validation. As artificial intelligence (AI) is being applied in proteomics, we must ensure that publicly available data are accurate and of high-quality to be used for downstream analyses and training models. With this motivation, our results are available in PRIDE (PXD071918), PeptideAtlas and UniProtKB, ensuring that this PTM data is FAIR and “AI-ready”.

## Graphical abstract

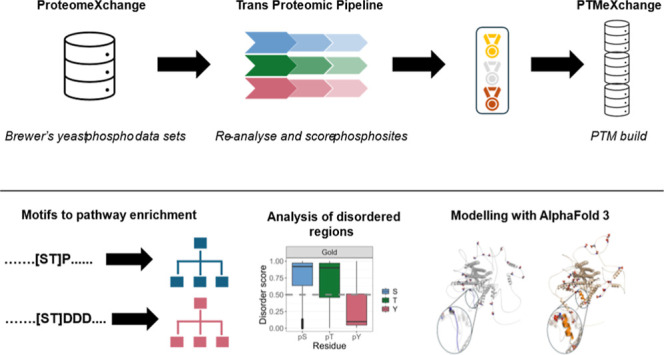

## Introduction

Cell signaling is vital for cells to coordinate activities and respond rapidly to changes in their environment. Reversible post-translational modifications (PTMs) are one mechanism by which cells implement such communication networks.^1^ Among the most studied PTMs is phosphorylation, which can act as a molecular switch, for example, activating or deactivating proteins via the activities of kinases and phosphatases.^2^ When these tightly regulated signaling pathways go awry, diseases may result, and as such, these pathways have become targets for the development of drugs.^3^ In this context, *Saccharomyces cerevisiae* (here referred to as “yeast”) has emerged as a popular model organism for studying phosphorylation since it is easy to culture, and many of its kinases and phosphatases have human orthologs.^4,5^ Furthermore, studying phosphorylation in yeast may also reveal insights into the phosphorylation networks of other eukaryotes due to the conservation of signaling components.^6^

Here, our first goal is to accurately identify phosphorylation sites (phosphosites) in yeast proteins and produce data outputs for the wider proteomics community, ensuring that PTM data is FAIR (Findable, Accessible, Interoperable, and Reusable).^7^ For context, previous phosphoproteome studies in yeast have identified between ∼30,000 and ∼45,000 phosphorylation sites,^4,8^ which is in line with a prior modeling estimation.^9^ Mass spectrometry (MS) is the dominant method for studying phosphosites on proteins as well as their localization. Briefly, this involves the enzymatic digestion of proteins (usually with trypsin) so that they are of a suitable mass to be analyzed by the mass spectrometer. Since PTMs (and phosphorylation in particular) may be of low abundance, samples are enriched for phosphorylated peptides using titanium dioxide, an immobilized metal affinity column, or antiphosphorylation antibodies. The peptides then enter the liquid chromatography-tandem mass spectrometry (LC–MS/MS) part of the workflow, where they undergo separation and ionization so that they can be detected and analyzed by the mass spectrometer. Then, the resulting MS data are searched against a protein sequence database. Here, the theoretical spectra (from an in silico digest of the database) are compared against the experimental spectra using a search algorithm. Finally, scores relating to the confidence of the peptide identification along with the localization of any PTMs are calculated.

The use of rigorous statistics for controlling the false discovery rate (FDR) and false localization rate (FLR) of PTMs is important to ensure that any data produced and shared with the wider community are accurate and useable. Most studies now control the FDR through the “target-decoy” approach,^10^ methods to control the FLR have been less frequently used.^11^ As such, it has been reported that a large number of false positive phosphorylation sites may exist within databases.^12^ Consequently, our group has published the pASTY method whereby an amino acid that cannot be modified, such as alanine for phosphorylation, is used as a decoy amino acid to calculate the global FLR across all the spectra within a data set.^11^ We have also adapted the method to recalculate the global FLR, taking into account redundant observations of the same peptidoform (i.e., peptide sequence and set of modifications carried) across multiple spectra within one study and across multiple studies.^13^ With the increased application of artificial intelligence (AI) approaches to proteomics data, it is vital to ensure that MS raw data is (re)­processed using rigorous statistical pipelines so that the input data for these models are of a high quality (very low error) and accompanied by an evidence trail so that downstream users can have confidence in any results derived from input data sets.^14^

Here, we present work produced from the PTMeXchange consortium (www.proteomexchange.org/ptmexchange), whereby we have reanalyzed phosphorylation-enriched data sets from brewer’s yeast (*Saccharomyces cerevisiae*) using our rigorous statistical pipeline for controlling FDR and FLR, as previously illustrated for phosphorylation in rice^15^ and *Plasmodium falciparum*,^16^ and also for other PTMs such as ubiquitination.^17^ We have then made the outputs available to the wider community by integrating the results into the widely used resources PeptideAtlas,^18^ PRIDE,^19^ and UniProt Knowledge Base (UniProtKB),^20^ thus giving the potential for further studies to explore the downstream biological consequences.

## Methods

### Data Set Selection and Curation

First, we selected and manually curated data sets in ProteomeXchange resources^21^ relevant for studying phosphorylation in *Saccharomyces cerevisiae*. We prioritized the inclusion of data sets which were produced using a Thermo Fisher Scientific instrument and generated in data-dependent acquisition mode (as these are straightforward to analyze using a robust open-source pipeline) and likely to be of high quality. Specifically, data sets with ≥20 raw files deposited in the repository and ≥5000 phosphosites reported in the original publication (if available) were considered. The raw files from each of the selected eight data sets which met these criteria were downloaded from the PRIDE repository:^22^ PXD000554,^23^ PXD012395,^8^ PXD013271,^24^ PXD019647,^25^ PXD021109,^26^ PXD028028,^27^ PXD035029,^4^ and PXD037381 ^28^ (Table 1).

**Table 1:** Overview of the Key Characteristics of the Data Sets Included in the Yeast Phospho-Build as Well as the Parameter Tolerances Used in the Comet Search

data set	instrument	count of RAW files	peptide mass tolerance (ppm)	fragment tolerance (Da)	number of phosphosites reported in original publication	quantification approach	conditions tested
PXD000554 ^23^	Q Exactive	53	10	0.02	8961	SILAC	mock treated (no rapamycin), 200 nM rapamycin for 1 h and 200 nM rapamycin for 3 h
PXD012395 ^8^	Q Exactive	811	10	0.02	30,902	SILAC	asynchronous, asynchronous and DNA damage, S-phase, G1-phase, G1 and DNA damage, G2/M-phase, G2/M and DNA damage, carbon limiting, and chymotryptic digest
PXD013271 ^24^	Q Exactive Plus/Q Exactive HF-X	234	10	0.02	36,643	SILAC	wildtype or knockout atg1Δ cells either treated with rapamycin (200 ng/mL for 30 min) or not treated with rapamycin
PXD019647 ^25^	Q Exactive HF	29	10	0.02	8611	SILAC	wildtype (WR209) and rts1Δ strains in exponential growth
PXD021109 ^26^	Q Exactive HF-X	132	10	0.02	35,401	SILAC	wildtype, sui2^S52A^ and gcn2Δ either treated with rapamycin (200 ng mL^–1^ for 30 min) or not treated with rapamycin
PXD028028 ^27^	Q Exactive HF-X	278	10	0.02	48,058	SILAC	wildtype, rim15Δ, yak1Δ, slt2Δ, and npr1Δeither treated with rapamycin (200 ng mL^–1^ for 30 min) or not treated with rapamycin
PXD035029 ^4^	Q Exactive/Orbitrap Eclipse/Orbitrap Exploris 480	80	10	0.02	36,405	label free	101 environmental and chemical perturbations. Most treatments were for 5 min
PXD037381 ^28^	Q Exactive HF-X/Orbitrap Exploris 480	240	10	0.02	40,547	SILAC/label free	SILAC - DMSO or 2NM-PP1 at 0.05% glucose for 5 min (medium-heavy) or 15 min (heavy) and, 2% glucose (light)
							label free - On bead in vitro kinase assays with either wildtype Snf1 or kinase-dead Snf1

### Search Database

The protein sequence database from *S. cerevisiae* (UP000002311) was downloaded (27/11/2023) from UniProtKB with the inclusion of isoforms. Reversed decoy and contaminant (cRAP proteins: https://www.thegpm.org/crap/) proteins were added to the database via FragPipe (https://fragpipe.nesvilab.org/).

### Preliminary Processing

First, we converted the raw files to mzML^29^ using ThermoRawFileParser^30^ and deployed two preliminary processing steps to learn the database search parameters for each data set. Here, we used FragPipe, which is powered by MSFragger, to run an open search whereby a wide tolerance was used when comparing the experimental spectra to the theoretical spectra.^31^ The results of FragPipe allowed us to detect any samples that had unexpected peptide/protein modifications or quantification reagents/labels that may be present, which may not have been fully or correctly specified by submitters when uploading the data sets to ProteomeXchange. We also used a tool called RunAssessor (https://github.com/edeutsch/RunAssessor),^59^ which outputs details on the counts of MS1 and MS2 spectra, iTRAQ or TMT labeling (if used), type of fragmentation, and mass spectrometer instrument used.

### FDR and FLR Pipelines

The bioinformatics pipeline has been explained in detail previously^11^ and uses tools included in the Trans Proteomic Pipeline (TPP) version 6.3.2.^32^ In the first step, the Comet search algorithm version 2021.01 rev0 ^33^ was used as the search engine. In all data sets carbamidomethylation was included as a fixed modification, and phosphorylation on STYA, methionine oxidation, and protein N-terminal acetylation were included as variable modifications. The inclusion of phosphorylation on alanine was part of the pASTY decoy method to calculate the FLR.^11^ Depending on the open search results per data set, additional variable modifications included SILAC labeling, pyro-glu from E, pyro-glu from QC, and deamidation (NQ). For all data sets, other selected parameters included a full trypsin digestion, the allowance of two missed cleavages, and a maximum of five variable modifications per peptide.

Table 1 contains a summary of the key characteristics and search tolerances used for the data sets. (Further details of the variable modifications and quantification approaches can be found in Data S1). The resulting pepXML files from the Comet search were then aggregated and processed using PeptideProphet (which provides peptide-spectrum match (PSM) statistics),^34^ iProphet (which provides peptidoform statistics),^35^ and PTMProphet (which provides PTM site localization statistics).^36^

The FDR and FLR open pipelines (mzidFLR, https://github.com/PGB-LIV/mzidFLR) were used for statistical analysis of the TPP output files.^11,13^ The hits to decoys were used to calculate the global FDR at the PSM level. A threshold of 1% FDR was used for filtering the PSM results. After the results were expanded to the site-based format (each row was a phosphosite on a peptide), they were filtered to retain phosphorylation modifications while discarding decoys and contaminants. The probability that a given site was correct in one spectrum was estimated by multiplying the PTM probability (PTM localization) by the PSM probability (peptide identification). The PSM site-based file was converted into a peptidoform site-based file, thereby reducing the redundancy introduced from multiple PSMs giving evidence for the same peptidoform. A binomial probability-based correction was applied to the PTM final score to account for PTM sites with fewer PSM counts being more likely to be false positives.^13^

Similarly, phosphorylated alanine residues were used as a decoy, since these are known to be incorrect localizations, to calculate the global FLR.^11^ When the results from each experiment were collapsed in a single PXD data set, the row with the lowest FLR for a peptidoform was retained.

Finally, we applied the Gold-Silver-Bronze (GSB) classification to combine the results across all data sets. It is crucial to control FLR inflation in this way since the number of false positives in the meta-analysis increases at a faster rate than the number of true positives.^13^ According to the GSB approach, a site is classified as Gold if it has <1% FLR and it was identified in at least n data sets. For Silver classifications, there must have been evidence for the site in at least m data sets, where *m* < *n*, with <1% FLR. Finally, Bronze encompassed all remaining sites at <5% FLR. With a build of eight data sets, we selected *n* = 2 and *m* = 1, following inspection of the resulting decoy hits in the final build. While Gold phosphosites are the most useful for circumstances where correct identification is integral, the Silver and Bronze categories may be important for other analyses and thus should not be disregarded. For each phosphosite, multiple protein mappings were considered as potentially correct, under the assumption that proteins sharing the same phosphorylation motif (or at least the same peptide sequence) are all likely to be phosphorylated. In each confidence category, the estimated FLR was calculated by multiplying the alanine count by the ratio of STY to A as calculated from peptidoforms across all data sets after collapsing for redundancy at the peptidoform level.

### Downstream Analysis of the Results

#### Motif and Pathway Enrichment Analysis

From the single mapping protein list of Gold STY phosphosites, 15mer peptides were generated and compared to a background of 15mer peptides centered around S or T from all identified peptides at 1% FDR. Motifs with S or T as the central residue that were significantly enriched in the data set were identified using rmotifx.^37^ We then repeated the analysis using a foreground of 15mers centered around S or T GSB sites. While the Gold set is important for analysis where strict identification of sites is required, the GSB set may be useful in identifying a higher number of significant motifs. However, it should be noted that the higher number of false positive sites in the GSB list compared to the Gold list may weaken the statistical power.

Similarly, as an adjacent analysis, we created a background of 15mer peptides with Y as the central residue (from all identified peptides at 1% FDR) to identify whether there were any significantly enriched tyrosine motifs in the tyrosine-centered foreground.

Motifs were grouped in the first instance according to the kinase motif classes which had previously been described (as proline-directed, acidic, basic, and other).^38^ Additionally, we further divided the “other” group based on the properties of the amino acids present in the motif. A binary decision tree was used to characterize the amino acids in the motifs, as follows: P at +1 (proline-directed), ≥5 E/D at +1 to +6 (acidic), R/K at −3 (basic), E/D at +1/+2 or +3 (acidic), ≥2 R/K at −6 to −1 (basic), any NQST residues, any DE residues, any KR residues, or other. For each motif class, the foreground sequences were filtered so that they contained only sequences that matched the motifs in the class. The sequences present in each class (with X’s used to denote gaps in the alignment) were visualized as an iceLogo^39^ with the reference set from rmotifx being used to compare amino acid frequencies at each position.

Pathway enrichment analysis was performed per motif class using the enrichGO­(OrgDb = org.Sc.sgd.db, keyType = “UNIPROT”, ont = “ALL”, pAdjustMethod = “BH”, pValueCutoff = 1, qvalueCutoff = 1) function in clusterProfiler^40^ for “ALL” gene ontologies (i.e., the three categories of terms: biological process, molecular function, and cellular component). The conversion of UniProt IDs to gene ontology (GO) IDs^41^ was performed using the org.Sc.sgd.db annotation database.^42^ In each case, the foreground proteins were compared to a background of all Gold S/T phosphoproteins to identify potentially enriched pathways relating to the motif class. The GO terms from enrichGO­() were filtered to retain only those that were significantly enriched (Benjamini–Hochberg adjusted *p*-value <0.01 and *q*-value <0.2). To collapse redundancy between GO terms, the simplify function with a cutoff of 0.6 was used. Finally, a heatmap of -log10 adjusted *p*-values generated with pheatmap^43^ was used to elucidate potential patterns in enriched GO terms across the motif classes for each GO term category.

#### Analysis of Disordered Regions

To understand the structural context of phosphorylation, we used metapredict V3 ^44^ to investigate the predicted degree of disorder of STY sites (using only those singly mapped to proteins to avoid bias) compared to all other STY sites in our search database (excluding contaminants and decoys). For each amino acid in the search database, metapredict assigned a score from 0 (ordered) to 1 (disordered). A threshold of 0.5 is used to distinguish between ordered and disordered proteins.^44^ For comparison, we performed a similar analysis using a curated set of Gold standard human phosphosites.^45^

#### Analysis Using AlphaFold 3

With the release of AlphaFold 3 (AF3) in 2024 ^46^, there was a significant advancement in the ability to model not only protein sequences with unprecedented accuracy but also structures of proteins containing PTMs (when compared to AlphaFold 2). Here, we curated a list of proteins from our GSB results that had at least 10 Gold phosphorylation sites (682 proteins). Each protein was then modeled twice using AF3 −first as a protein sequence alone (no PTM model) and second with the addition of the Gold sites (PTM model), thus generating a total of 1364 models. For each model, we defined the secondary structural elements within the output generated from AF3 using the Dictionary of Secondary Structure in Proteins (DSSP) software.^47^ The secondary structural content in the PTM versus no-PTM models was compared along with the pLDDT confidence scores: these scores capture confidence in local prediction accuracy on a scale from 0 to 100. Specifically, comparisons were made in the overall counts of amino acids in each structural category as well as in the length (amino acid count) of secondary structural elements at phosphosites. The latter of these was collapsed such that if a structural element contained more than one phosphosite, it was only included once in the results. For the confidence analysis, the mean pLDDT score was calculated from the atom scores for serine and threonine residues in both the PTM and no PTM models.

Finally, the impact of combinations of close proximity phosphosites on the induction of structural changes in a protein was briefly explored in the heavily phosphorylated Transposon Ty1-PL Gag-Pol polyprotein (Q12414). Here, we modeled the first 50 amino acids of the protein five times in each of the following states: phosphorylated at S3, phosphorylated at S7, phosphorylated at S3 and S7 and nonphosphorylated.

## Results and Discussion

We reanalyzed eight public yeast phospho-enriched data sets to get an updated view on the yeast phospho-proteome. We used an open pipeline that includes Comet, TPP, and the mzidFLR methodology, as applied previously and described in “Methods”. Figure 1A summarizes the workflow used. Since redundancy may exist from multiple PSMs giving evidence for the same peptidoform, counts are summarized at the peptidoform-site level at 1% FLR and 5% FLR as well as an overall count (Figure 1B). Here, redundancy was also removed at the data set level, where multiple experiments provided evidence for the same peptidoform.

**Figure 1:**
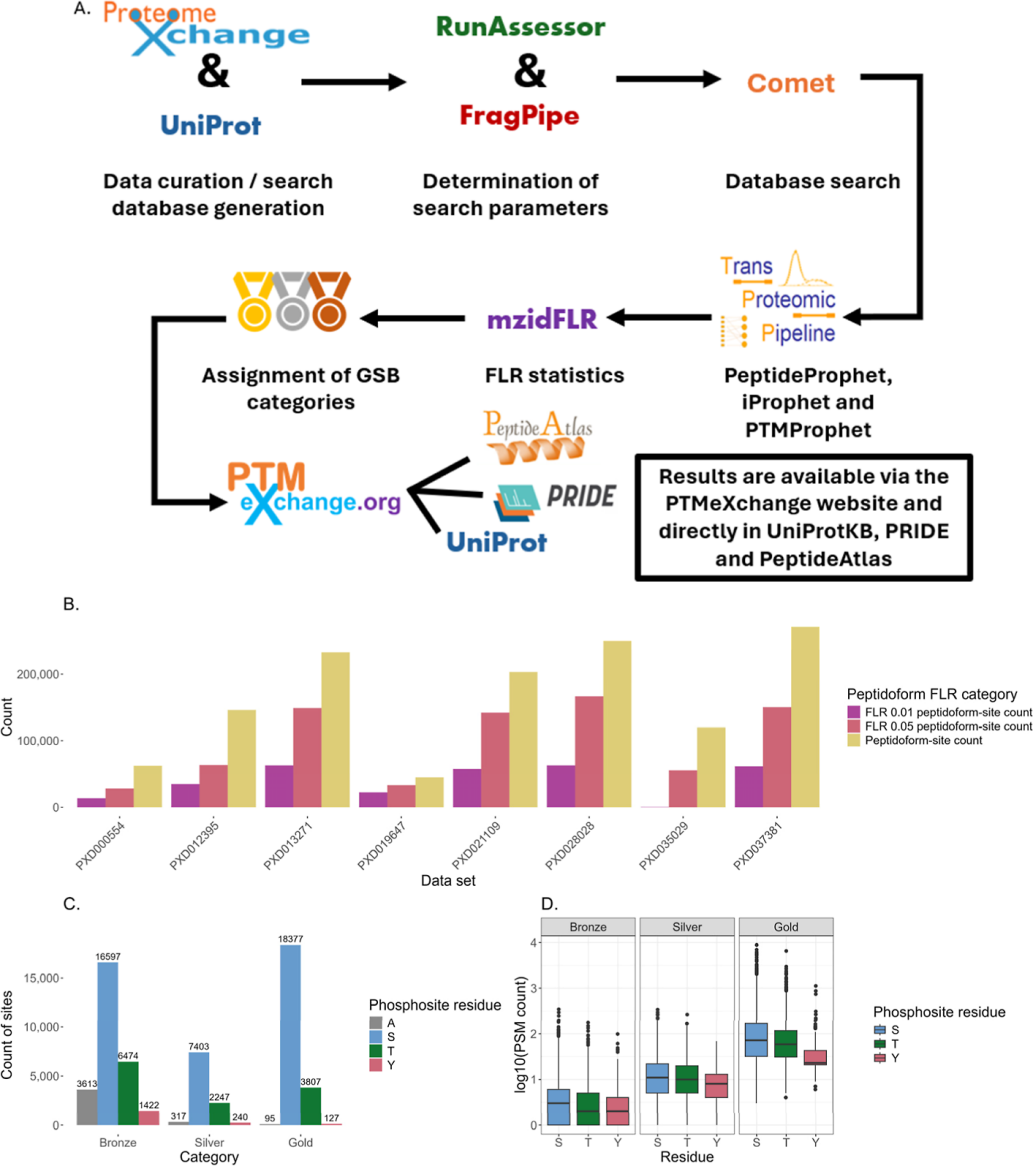
Summary of the yeast phosphorylation build. (A) Overview of the workflow used for the yeast phosphorylation build. (B) Phosphosite counts after the peptidoform collapse for removing redundancy at 1% and 5% false localization rate (FLR) levels per data set. (C) Phosphosite counts on S, T, and Y compared to the decoy amino acid (A, alanine) in the Gold, Silver, and Bronze (GSB) confidence categories. Here, multiple mappings of the peptide to proteins have been considered. (D) Peptide spectrum match (PSM) counts per phosphosite for S, T, and Y in each of the Gold, Silver, and Bronze confidence categories. The whiskers display the range of values up to 1.5 * interquartile range from the first and third quartiles. Any data points which fall outside of the whiskers are deemed to be outliers.

### Gold-Silver-Bronze Classification

To validate our list of phosphosites in yeast, we combined the analysis from all individual data sets and categorized each protein site depending on the overall confidence of the identification (Figure 1C). Briefly, phosphosites at <5% FLR were categorized as Gold, Silver, or Bronze based on the FLR threshold and the number of data sets they were identified in. Gold sites were those identified at <1% FLR in at least two data sets, Silver sites were those identified at <1% FLR in one data set, and Bronze sites were all other sites identified at <5% FLR. Here, hits to decoys were retained to compare the relative numbers of alanine sites to S, T, and Y sites in each confidence category. As such, the alanine count in the Gold category was relatively low (*n* = 95) and thus the estimated FLR (alanine count multiplied by the STY/A ratio) was also low when compared to the Silver (*n* = 317) and Bronze (*n* = 3613) categories, which contained a relatively higher number of alanine residues and thus a higher FLR.

Overall, when removing the decoy hits and accounting for multiple mappings of a peptidoform to a protein, we report a total of 56,694 phosphosites on S, T, and Y, with 22,311 (39.4%) classified as Gold, 9890 (17.4%) classified as Silver, and 24,493 (43.2%) classified as Bronze. Among the Gold sites, 82% occurred on serine, 17% occurred on threonine, and just 1% occurred on tyrosine. This low proportion of phosphotyrosine is similar to figures reported in other studies^48^ suggesting tyrosine phosphorylation is scarce or has low site occupancy on yeast proteins.^4^ In the Silver set, the distribution of sites was 75%, 23%, and 2% for serine, threonine, and tyrosine, respectively. Finally, for the Bronze set, 68% of sites occurred on serine, 26% were on threonine, and 6% were on tyrosine. The increase in the distribution of sites on tyrosine residues from Gold to Silver to Bronze likely reflects the quality of data in terms of the increasing numbers of false positives, as false positives will be approximately equally split among S, T, and Y. Single and multiple mappings of the phosphosites to proteins can be found in Datas S2 and S3, respectively.

Finally, we quantified the abundance of sites in terms of the count of PSMs supporting phosphosites in each of the confidence categories (Figure 1D). On a per residue basis, there was a graded increase in the average number of PSMs that supported a phosphosite. Thus, phosphosites that were classified in the lowest confidence category (Bronze) tended to have a lower number of supporting PSMs than those classified in the highest confidence category (Gold).

### Downstream Analysis

#### Motif and Pathway Enrichment Analysis

We next investigated our list of (single mapping to proteins) Gold phosphosites since this is a high-quality set of identifications, using motif and pathway analysis. Rmotifx was used to identify 55 motifs that were significantly enriched and had an S or T as the central residue (Table 2). In terms of the motif groups, 5 were proline-directed, 16 were basophilic, 9 were acidophilic, and the rest were categorized in the various “other” subcategories (Data S4; see Data S5 for a description of the columns). Furthermore, when rmotix was run using a foreground of GSB S/T phosphosites, 70 significantly enriched motifs were identified (Data S6). When selecting the top 30 motifs in terms of foreground matches in the GSB and Gold results, there was an overlap of 21 motifs (Figure S1).

**Table 2:** Summary of Motif Patterns (Identified Using Rmotifx) in Each Class and Number of Proteins Containing at Least One Instance of Each Class of Motif^a^

motif class	motifs	number of motifs	count of proteins
proline-directed	.....P.[ST]P......, .......[ST]P..S..., ....R..[ST]P......, .......[ST]P.K...., .......[ST]P......	5	1831
acidophilic	.......[ST]D.E...., .......[ST]DDD...., .......[ST]D.D...., .......[ST]E.E...., .......[ST].DD...., .......[ST]QE....., .....N.[ST].E....., .......[ST].DE...., .......[ST].E.....	9	1587
basophilic	....RR.[ST]......., ....RS.[ST]......., ....R..[ST]..S...., ....KR.[ST]......., ...SR..[ST]......., ..L.K..[ST]......., ....RN.[ST]......., ....R..[ST].E....., ....R.N[ST]......., ....K..[ST]...L..., ....R..[ST]..N...., ....R..[ST]..D...., ....K.N[ST]......., ....R.G[ST]......., ....R..[ST]......., ....K..[ST].......	16	1971
DE residues	....D.D[ST]......., ...DD..[ST]......., ....D..[ST]......., ....E.D[ST]......., ....E..[ST]......., ......D[ST].......	6	1917
NQST residues	.R..S..[ST]......., ....S..[ST]L......, .....N.[ST]Q......, ....S..[ST]I......, ....S..[ST]......., .....RN[ST]......., ......N[ST]......., .......[ST]Q......, .....N.[ST]......., ....T..[ST]......., .......[ST]...SP.., .......[ST].S.....	12	2438
KR residues	.....R.[ST]......., ......K[ST].......	2	1341
other	......G[ST]......., ....G..[ST]......., ....A..[ST]......., .......[ST]F......, ......P[ST].......	5	2029

a The motifs are written such that any residue within the square brackets is permissible at that given position, and “.” denotes that any amino acid is allowed. Thus, each motif has either S or T as the central residue with seven amino acids on either side

Conversely, there were no significantly enriched motifs in the 15mers with tyrosine as the central residue.

For each motif class, the top five significantly enriched GO terms in each of the ontology categories are displayed in the dot plot figures along with the corresponding iceLogo in Figures 2 and S2. A comparison between enriched GO terms in the different motif classes is displayed in Figures S3–S5. Compared to a background of Gold S/T phosphoproteins, the following GO terms were enriched in the proline-directed class: intracellular signal transduction (GO:0035556), cytoskeleton organization (GO:0007010) (biological processes), and transcription regulator activity (GO:0140110) (molecular function). Previous studies investigating the osmotic stress response in yeast identified the enrichment of similar terms related to signaling and the cytoskeleton.^49–51^ Across all motif classes, there was a significant enrichment of the “site of polarized growth” (GO:0030427) GO term within the cellular component ontology. Finally, there was significant enrichment of GO terms relating to protein kinases (GO:0004672, GO:0106310, and GO:0016301) in the molecular function ontology terms in several of the motif classes, as expected since many kinases have their activity regulated by phosphosites (through autophosphorylation or regulation by other kinases). This pathway enrichment analysis was performed using clusterProfiler, and the full results for each motif class before statistical thresholding are included in Data S7.

**Figure 2:**
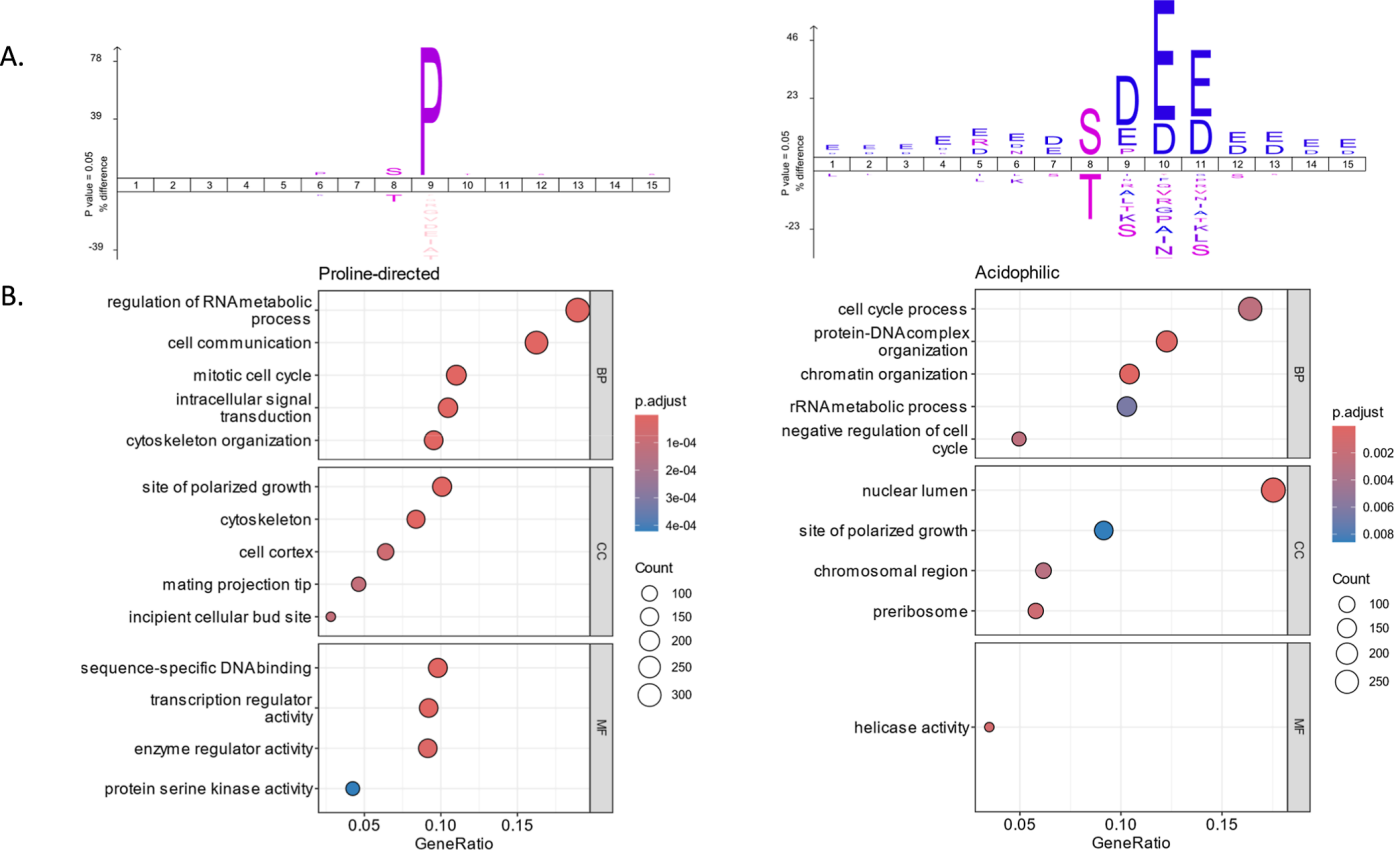
Motif and pathway enrichment analysis for the proline-directed (left) and acidophilic (right) motif classes. (A) iceLogos of motif classes to show the representation of amino acids at a given position either side of the central residue (serine or threonine). (B) Enriched pathways, as derived from clusterProfiler, in the group of proteins containing a motif from the given motif class.

#### Analysis of Disordered Regions

In terms of the structural context, we explored whether the phosphosites tended to occur on ordered or disordered residues in proteins. Here, phosphosites on Y (median score = 0.19) tended to occur in ordered regions, while phosphosites on S (median score = 0.90) and T (median score = 0.84) tended to occur in disordered regions of the protein, corroborating previous studies.^4,52,53^ This pattern was most pronounced in the Gold data set (Figure 3). In comparison, nonphosphorylated STY sites (median scores: *S* = 0.19, *T* = 0.15, and *Y* = 0.09) all tended to occur in ordered regions of proteins (Figure 3). Previous work by our group^45^ has indicated that similar patterns are observed in Gold-standard human STY phosphosites in comparison to nonphospho STY sites (Figure S6).

**Figure 3:**
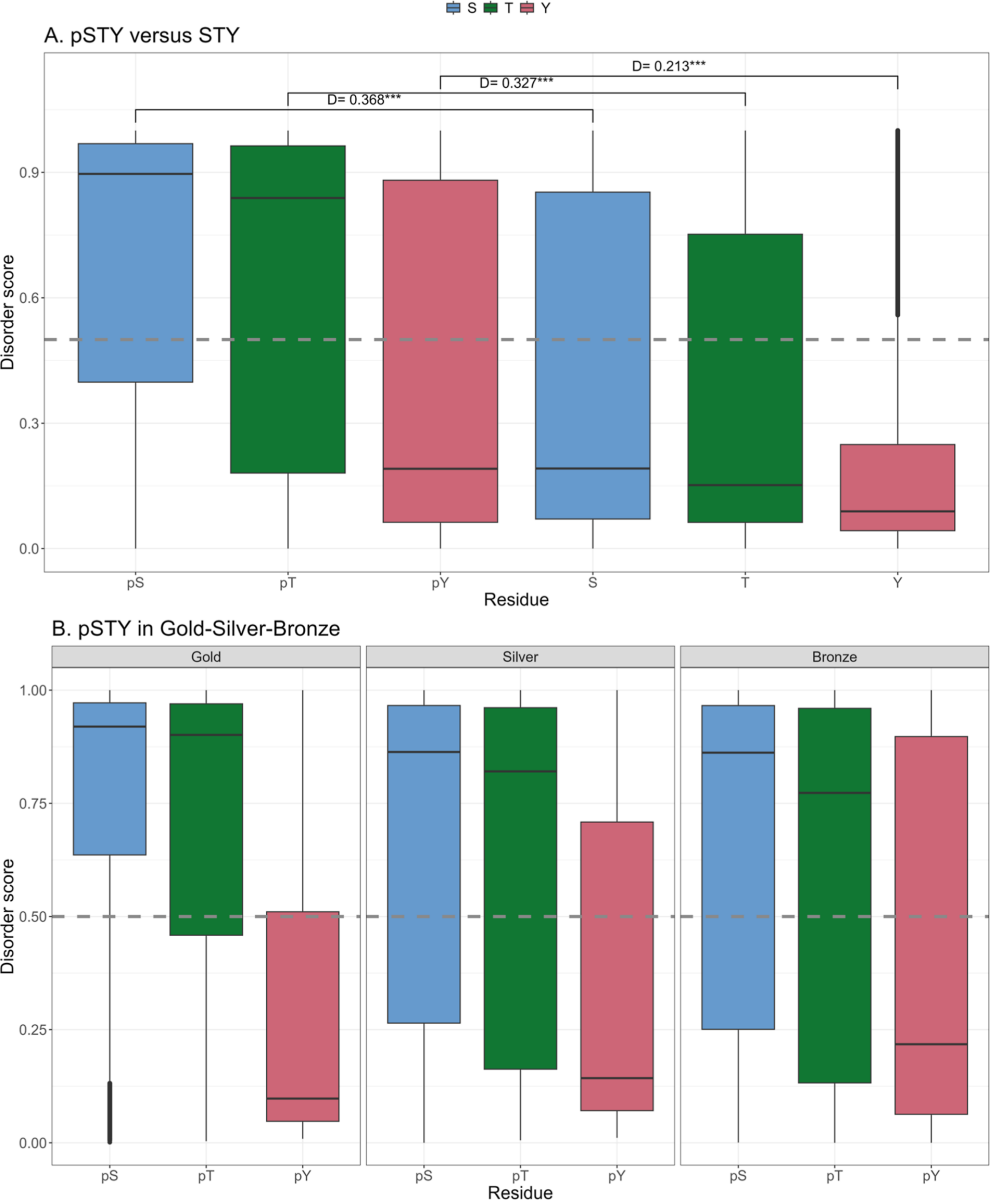
Disorder predictions from metapredict suggest that phosphorylated serine and threonine residues tend to occur in disordered regions of proteins, while phosphorylated tyrosine residues occur in ordered regions. (A) Disorder in phosphorylated STY sites (single mapping) compared to all other STY sites in the yeast database (from 0 (ordered) to 1 (disordered)). A two-sample Kolmogorov–Smirnov test was performed to compare the distribution of disorder scores (*** = *p*-value <0.001) in the phosphorylated and the nonphosphorylated state for each residue. (B) Disorder analysis for reported phosphorylated STY sites in yeast by Gold-Silver-Bronze confidence category. The dashed line represents the threshold of 0.5, which is used to distinguish between ordered and disordered regions of a protein in metapredict V3. The whiskers display the range of values up to 1.5 * interquartile range from the first and third quartiles. Any data points which fall outside of the whiskers are deemed to be outliers.

#### Analysis Using AlphaFold 3

We have utilized the capabilities of AF3, the first version of AF that can consider PTMs such as phosphosites when modeling the structure of proteins, to model 682 proteins both as the protein sequence alone as well as with their phosphosites in the “Gold” category. By using our high-quality set of phosphosites, this makes for an ideal comparison of the structure of yeast proteins in the phosphorylated and nonphosphorylated states.

Preliminary results suggest that there was a significant association between the type of secondary structure present and whether phosphosites were included in the AF model (χ^2^ = 5975.1, df = 7, *p*-value= < 0.001). Specifically, there were instances whereby phosphorylation induces secondary structure in the form of alpha helices into the protein structure (Figure 4A) when modeling with phosphosites is performed, compared to when modeling is performed without phosphorylated residues. As well as a significant increase in the main α helix category (Chi-square *p*-value with Bonferroni correction <0.001), there were also increases in the rarer 3–10 helix and left-handed helix along with the bend and turn categories. Conversely, there was a significant decrease in loops in the phosphorylated protein structure models.

**Figure 4:**
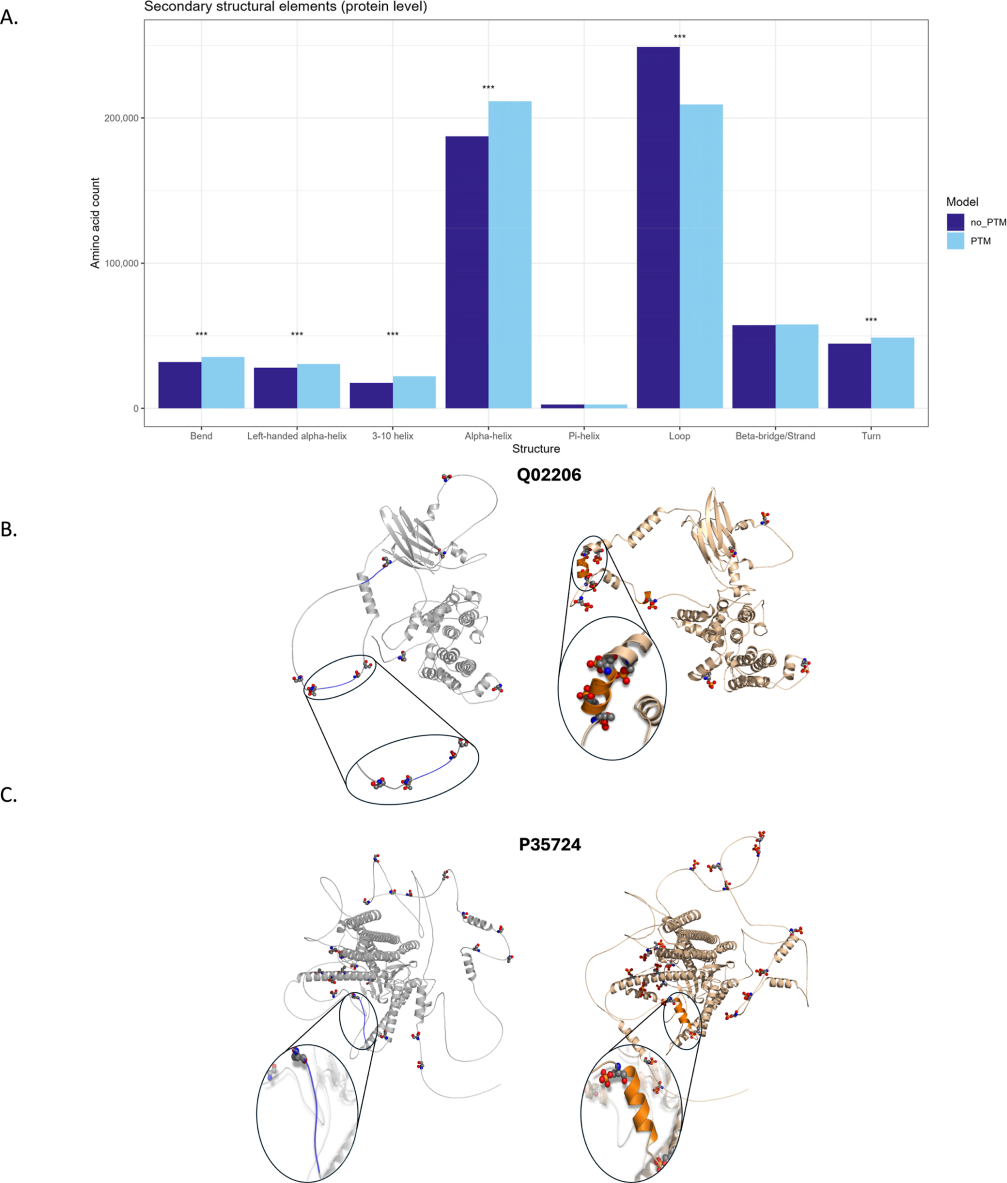
Summary of structural changes associated with phosphorylation in all proteins modeled (A) as well as in two representative proteins (B,C). (A) Amino acid count in secondary structure categories in yeast proteins modeled as the protein sequence alone (model = no_PTM) or containing their “Gold” phosphosites (model = PTM). Particularly, there was an increase in α helix and a decrease in loops at the protein level (Chi-square with Bonferroni correction, *** denotes a *p*-value <0.001). (B) Chromatin structure-remodeling complex subunit RSC4 (Q02206, phosphosites: residues 15, 199, 371, 396, 405, 406, 413, 416, 545, and 567) and (C) Manganese resistance protein MNR2 (P35724, phosphosite: residues 16, 21, 93, 104, 114, 117, 129, 152, 169, 175, 182, 383, 457, 461, 463, 472, 571, 576, 582, 612, 631, and 794) are shown in their nonphosphorylated (left) and phosphorylated (right) states following structural alignment. Regions that undergo phosphorylation-associated conformational rearrangement are highlighted (Q02206: residues 407–412 and 372–375 and P35724: residues 613–621), with the nonphosphorylated forms shown in blue and the phosphorylated forms shown in orange, while the surrounding structure is displayed as a pastel cartoon (gray for nonphosphorylated and wheat for phosphorylated). Phosphorylated residues are shown as sticks and spheres, colored by atom type (C: gray, O: red, N: blue, and P: orange).

**Figure 5:**
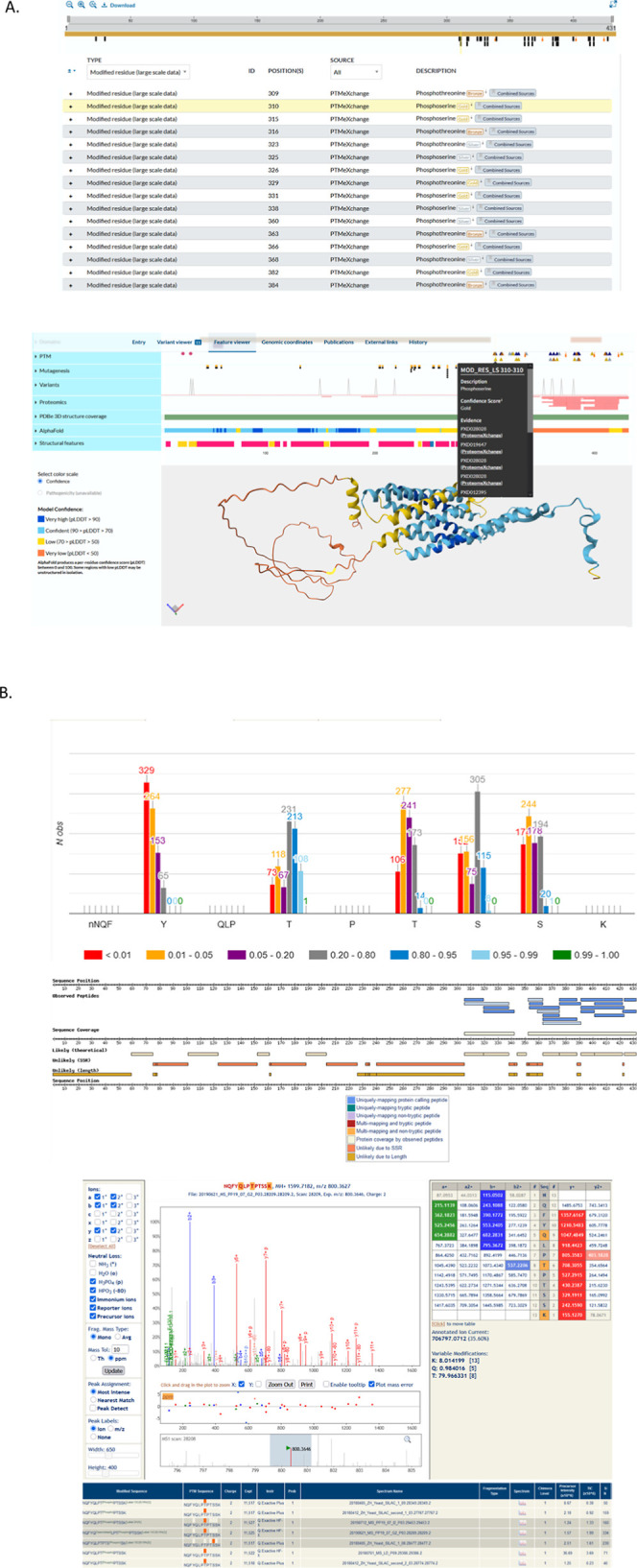
Data availability of the yeast phosphorylation build in UniProtKB and PeptideAtlas. (A) Example of a protein (pheromone alpha factor receptor, D6VTK4) whereby we report evidence for several phosphosites at the Gold, Silver, and Bronze confidence levels in UniProtKB. (B) Visualization of D6VTK4 in PeptideAtlas (PeptideAtlas peptide accession: PAp01013435).

We then moved from exploring the structural changes across all the proteins modeled to focus on two examples (Figure 4). In proteins Chromatin structure-remodeling complex subunit RSC4 (Q02206) and Manganese resistance protein MNR2 (P35724), there was an induction of α helix. In RSC4, the induced helix is closest to the RSC4 Ig-like domain, although it sits on a loop rather than forming an additional part of the domain, giving a hypothesis that it could be altering protein–protein interactions or complex formation. In MNR2, the induced helix sits proximal to the main domain (Alr1p-like subfamily) and thus provides a hypothesis that phosphorylation creates a structural change affecting its transporter function. We also explored the impact of phosphosites in close proximity to each other for a third heavily phosphorylated example protein (Transposon Ty1-PL Gag-Pol polyprotein; Q12414), which can be found in Figures S7 and S8, respectively. The results indicate that alpha helices are induced to form with only a single phosphosite added to the model for a given region and that multiple proximal phosphosites are not necessary to induce helix formation.

We next profiled the full set of AF3 models with and without phosphosites. The median length of the α helix encompassing the phosphosites tended to be shorter in the PTM model (13 amino acids (interquartile range = 8–21 amino acids)) compared to the no-PTM model (15 amino acids (interquartile range = 10–26 amino acids)) (Figure S9). However, the confidence, in terms of pLDDT score, with which these short helices are being predicted at the PTM site of serine and threonine residues is low (Figure S10, blue box) in the PTM model compared to that of the no-PTM model. This result is contrary to what might be expected since pLDDT scores of less than 50 are thought to correspond to regions of disorder rather than to secondary structure.^54^ Thus, further studies are required to understand whether such findings are biologically meaningful or rather an artifact of the AF3 model. For example, the location of the phosphosites in relation to the α helix could be explored. When phosphorylation occurs at the N-terminus of an α helix, it has been suggested that this is a stabilization mechanism^55^ and thus may align with the findings discussed here. Additionally, further validation of the use of AF3 to predict protein structures containing phosphorylation sites could be explored through comparison with experimental structures deposited after the training of AF3. This may be an important point to explore since the predictions here could reflect biases or artifacts in the data used in the training of AF3, particularly if there were a limited number of phosphorylated structures. All models generated are available at: 10.5281/zenodo.17737612.

## Conclusions

We present here the yeast “phosphosite build”, a meta-analysis of publicly available phosphoproteomics data sets in *Saccharomyces cerevisiae* that have been reanalyzed using open analysis pipelines. Briefly, this involved a database search step using Comet, calculation of FDR statistics relating to PSMs, peptidoforms, and PTM sites using modules in the TPP, and, finally, calculation of the global FLR using alanine as a decoy amino acid. We then classified sites into Gold-Silver-Bronze confidence categories. While we have focused on the utility of the high-confidence Gold sites, it should be noted that the Silver and Bronze sets may also be useful for some purposes, though not where strict identifications are required.

We paid particular attention to ensuring that the outputs of the study met FAIR guidelines. First, we have implemented the pASTY method for calculation of the global FLR. This step is often neglected in PTM studies, which may lead to a high number of false positive identifications being reported in the results. Thus, by strictly implementing FLR thresholds, we are ensuring that our outputs are of a high quality, which is important for downstream applications, and ensuring that PTM data is “AI-ready”. Second, to ensure outputs are easily accessible to the community, the results have been made available in PeptideAtlas, UniProtKB, and PRIDE. Along with this, a full evidence trail is available for exploration via Universal Spectrum Identifiers (USIs).

With 56,694 phosphosites, of which 22,311 fall in our highest confidence category (Gold), now accessible for exploration on various platforms, we envisage several avenues for downstream analysis and investigation by researchers. For example, as we have started to explore here, AF3 holds much potential in the sphere of structural biology and understanding the location of PTMs in a protein. Thus, our list of highly confident phosphosites could be used to explore the validity of AF3 predictions when residues are phosphorylated. Additionally, phospho-deficient strains created by mutating the phosphosite amino acid to an alanine could be produced. The functional importance of the phosphosite can then be evaluated depending on its response to stress perturbations in comparison to a library of gene knockouts. Similar work has been carried out previously for ∼470 phospho mutants, but we provide here the opportunity for an even larger-scale analysis in the order of thousands of phosphosites.^58^

Ultimately, our outputs are helping to ensure PTM data are FAIR and of high-quality as AI is increasingly being applied in the field of proteomics.

## Supplementary Material

















## Data Availability

The *Saccharomyces cerevisiae* “phosphosite build” has been produced as part of a project by the PTMeXchange Consortium to reanalyze high-quality PTM mass spectrometry data sets using open analysis pipelines and to make all results available to the community. First, phosphosites from the GSB results can be explored in UniProtKB under the “Entry” and “Feature viewer” pages (visible on https://www.uniprot.org/proteomes/UP000002311). With respect to the latter, phosphosites can be visualized on AlphaFold 2 predicted protein structures (Figure 5A). Here, the GSB category assigned to the site can be viewed as well as the originating data set identification accessions for the evidence. Second, the build has been released within PeptideAtlas for detailed investigation of the evidence available for a site at the protein, peptidoform, and spectrum level (https://peptideatlas.org/builds/yeast/phospho/) (Figure 5B). Finally, tab-separated files (per data set for both PSM and peptidoform level evidence), mzIdentML files,^56^ and the search database have been deposited in PRIDE under PXD071918 and 10.6019/PXD071918. In these resources, the evidence for each site is encoded as a Universal Spectrum Identifier (USI),^57^ which enables the associated spectrum for a site to be explored via https://proteomecentral.proteomexchange.org/usi/or https://www.ebi.ac.uk/pride/archive/usi. The code for the downstream analysis can be found on GitHub at: https://github.com/PGB-LIV/yeast_phospho. All AF3 models generated can be found at 10.5281/zenodo.17737612.

## Notes

Overlap of enriched motifs in Gold and Gold-Silver-Bronze rmotifx results; motif and pathway enrichment analysis for all kinase motif classes; heatmap of enriched gene ontology (GO) terms in the biological process category in the motif classes; heatmap of enriched GO terms in the cellular component category in the motif classes; heatmap of enriched GO terms in the molecular function category in motif classes; disorder scores for Gold standard pSTY sites compared to all STY sites in humans; transposon Ty1-PL Gag-Pol polyprotein (Q12414) is shown in its nonphosphorylated and phosphorylated states following structural alignment; structural analysis of AlphaFold 3 predictions for the first 50 residues of Transposon Ty1-PL Gag-Pol polyprotein (Q12414) across variable phosphorylation states; length of secondary structure encompassing the phosphorylation site when the protein is modeled containing its “Gold” phosphosites (model = PTM_phospho) compared to when the protein is modeled considering the amino acid sequence alone (model = no_PTM_phospho); pLDDT score of serine and threonine residues at the secondary structure elements accounting for sites identified as “Gold” phosphosites, as well as whether the protein has been modeled as the protein sequence alone or with the addition of the “Gold” phosphosites (PDF)
Search parameters used in Comet for each experiment (XLSX)
List of Gold-Silver-Bronze phosphorylation sites (residue and protein position) accounting for single mapping to proteins (CSV)
List of Gold-Silver-Bronze phosphorylation sites (residue and protein position) accounting for multiple mappings to proteins (CSV)
Motifs found in the Gold category (CSV)
Description of the columns in the rmotifx and clusterProfiler results (XLSX)
Motifs found in the Gold-Silver-Bronze input (CSV)
ClusterProfiler gene ontology enrichment results for all motif classes (before statistical thresholding) (CSV)
