# Supplementary material for: An AI-Ready Phosphorylation Meta-Analysis for Saccharomyces cerevisiae
Source: J Proteome Res. 2026 May 4;25(6):2915–26. doi: 10.1021/acs.jproteome.5c01263 (PMC13247961; doi:10.1021/acs.jproteome.5c01263)
Supplement: Supplementary file 1 [file pr5c01263_si_001.pdf]

An AI-Ready phosphorylation meta-analysis for *Saccharomyces cerevisiae*

Ellen L Boswell[1], Kerry A Ramsbottom[1], Jun Fan[2], Yasset Perez-Riverol[2], Emily H Bowler-Barnett[2], Zhi Sun[3], Shahram Mesdaghi[1], Daniel J Rigden[1], Maria J Martin[2], Eric W Deutsch[3], Juan Antonio Vizcaíno[2] and Andrew R Jones[1*]

[1] Institute of Systems, Molecular and Integrative Biology, University of Liverpool, Liverpool L69 7BE, United Kingdom

[2] European Molecular Biology Laboratory, EMBL-European Bioinformatics Institute (EMBL-EBI), Hinxton, Cambridge CB10 1SD, United Kingdom

[3] Institute for Systems Biology, Seattle, Washington 98109, United States

* Corresponding author email: Andrew.Jones@liverpool.ac.uk

Supplementary information

File	Location
Figure S1. Overlap of enriched motifs in the Gold and Gold-Silver-Bronze rmotifx results. The top 30 motifs in terms of foreground matches have been selected in both cases.	Page 4
Figure S2. Motif and pathway enrichment analysis for all kinase motif classes.	Pages 5 - 8
Figure S3. Heatmap of enriched gene ontology (GO) terms in the biological process category in the motif classes (up to the 'top 25' most significant terms). Gene counts and significance (*) for the GO term in each class are displayed.	Page 9
Figure S4. Heatmap of enriched GO terms in the cellular component category in the motif classes (up to the 'top 25' most significant terms). Gene counts and significance (*) for the GO term in each class are displayed.	Page 10
Figure S5. Heatmap of enriched GO terms in the molecular function category in motif classes (up to the 'top 25' most significant terms). Gene counts and significance (*) for the GO term in each class are displayed.	Page 11
Figure S6. Disorder scores for gold standard pSTY sites compared to all STY sites in humans as analysed in (45).	Page 12
Figure S7. Transposon Ty1-PL Gag-Pol polyprotein (Q12414) is shown in its non-phosphorylated and phosphorylated states following structural alignment.	Page 14
Figure S8. Structural Analysis of AlphaFold 3 predictions for the first 50 residues of Transposon Ty1-PL Gag-Pol polyprotein (Q12414) across variable phosphorylation states.	Page 15
Figure S9. Length of secondary structure (amino acids) encompassing the phosphorylation site when the protein is modelled containing its 'Gold' phosphosites (model= PTM_phospho) compared to when the protein is modelled	Page 17

considering the amino acid sequence alone (model= no_PTM_phospho).	
Figure S10. pLDDT score of serine and threonine residues at the secondary structure elements accounting for sites identified as 'Gold' phosphosites (green and blue), as well as whether the protein has been modelled as the protein sequence alone (green and grey), or with the addition of the 'Gold' phosphosites (purple and blue).	Page 17
Data S1. Search parameters used in Comet for each experiment.	Submitted separately
Data S2. List of Gold-Silver-Bronze phosphorylation sites (residue and protein position) accounting for single mapping to proteins.	Submitted separately
Data S3. List of Gold-Silver-Bronze phosphorylation sites (residue and protein position) accounting for multiple mappings to proteins.	Submitted separately
Data S4. Motifs found in the Gold category (rmotifx results).	Submitted separately
Data S5. Description of the columns in the rmotifx and clusterProfiler results.	Submitted separately
Data S6. Motifs found in the Gold-Silver-Bronze input (rmotifx results).	Submitted separately
Data S7. clusterProfiler gene ontology enrichment results for all motif classes (before statistical thresholding).	Submitted separately

Motif and pathway enrichment analysis

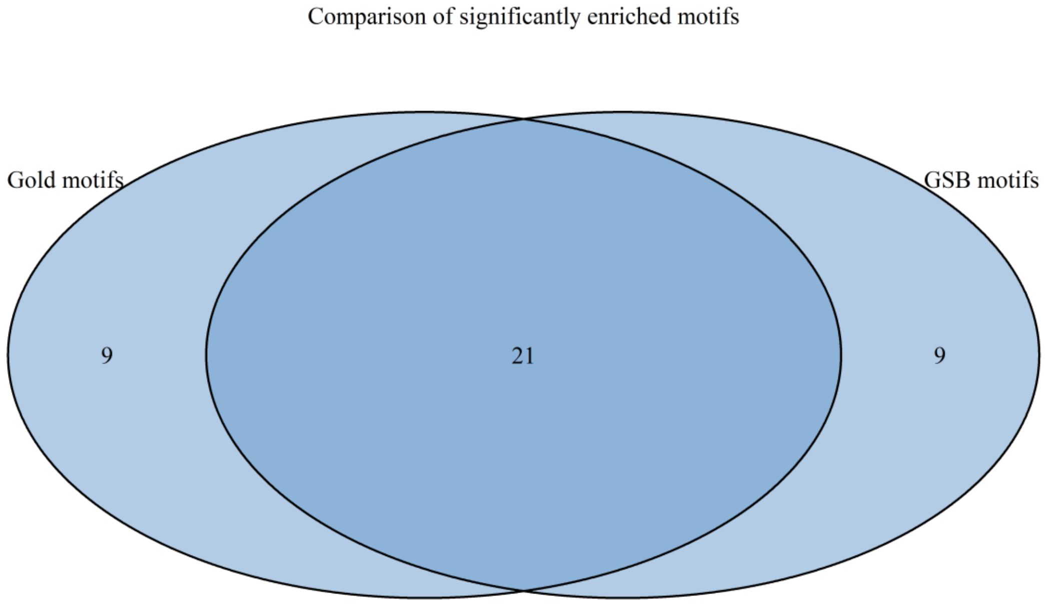

Figure S1. Overlap of enriched motifs in the Gold and Gold-Silver-Bronze rmotifx results. The top 30 motifs in terms of foreground matches have been selected in both cases.

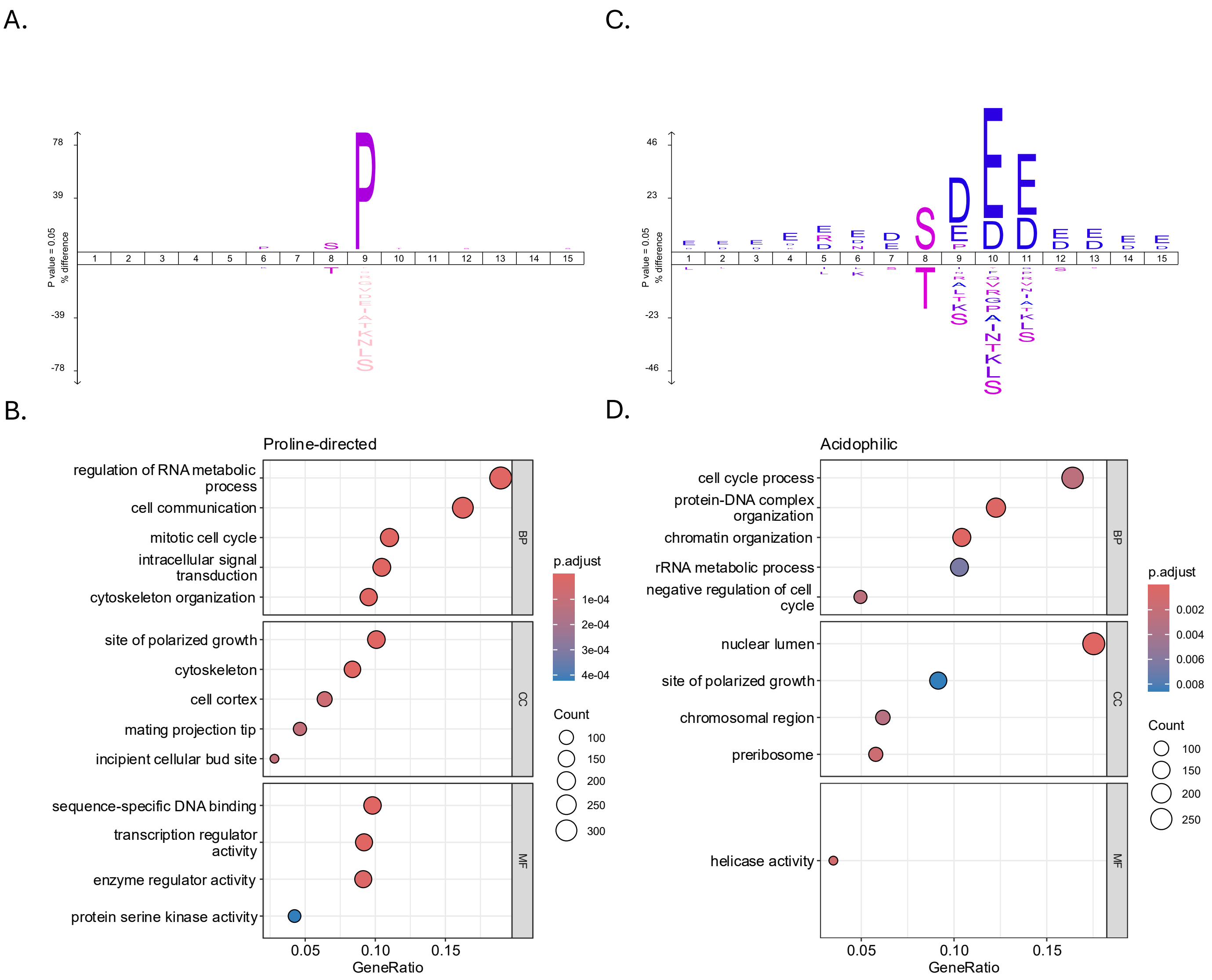
A.
C.
B.
D.
P value = 0.05
% difference
78
39
-39
-78
46
23
-23
-46
Proline-directed
regulation of RNA metabolic process
cell communication
mitotic cell cycle
intracellular signal transduction
cytoskeleton organization
site of polarized growth
cytoskeleton
cell cortex
mating projection tip
incipient cellular bud site
sequence-specific DNA binding
transcription regulator activity
enzyme regulator activity
protein serine kinase activity
BP
CC
MF
0.05
0.10
0.15
GeneRatio
p.adjust
1e-04
2e-04
3e-04
4e-04
Count
100
150
200
250
300
Acidophilic
cell cycle process
protein-DNA complex organization
chromatin organization
rRNA metabolic process
negative regulation of cell cycle
nuclear lumen
site of polarized growth
chromosomal region
preribosome
helicase activity
p.adjust
0.002
0.004
0.006
0.008
Count
100
150
200
250

E.

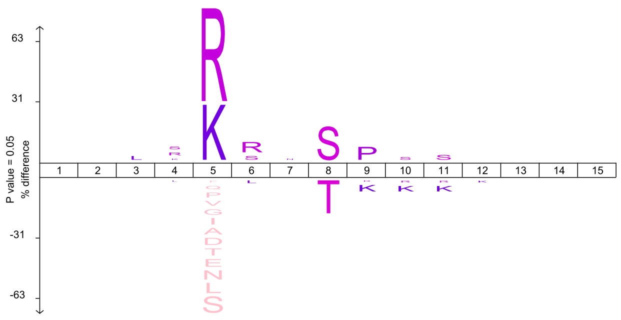

P value = 0.05
% difference
63
31
-31
-63
1 2 3 4 5 6 7 8 9 10 11 12 13 14 15

G.

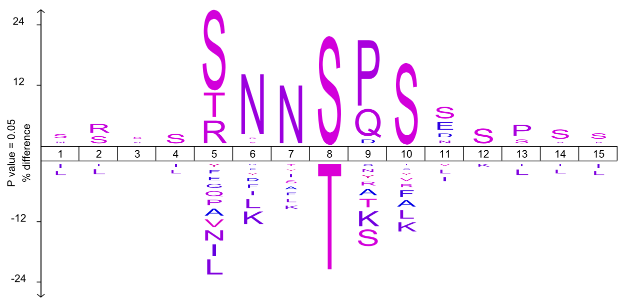

P value = 0.05
% difference
24
12
-12
-24
1 2 3 4 5 6 7 8 9 10 11 12 13 14 15

F.

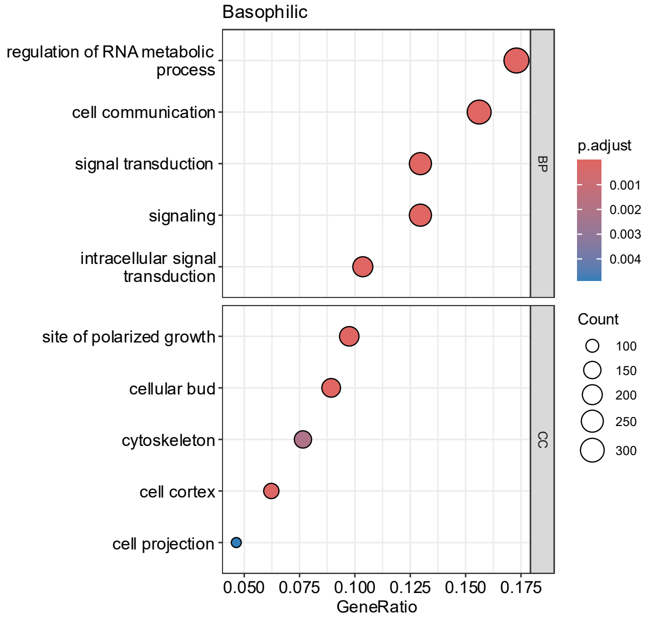

Basophilic
regulation of RNA metabolic process
cell communication
signal transduction
signaling
intracellular signal transduction
site of polarized growth
cellular bud
cytoskeleton
cell cortex
cell projection
BP
CC
p.adjust
0.001
0.002
0.003
0.004
Count
100
150
200
250
300
0.050 0.075 0.100 0.125 0.150 0.175
GeneRatio

H.

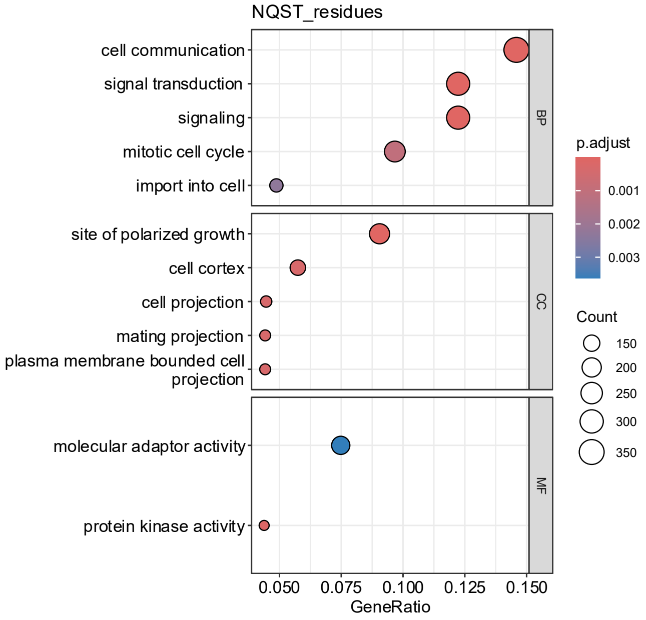

NQST_residues
cell communication
signal transduction
signaling
mitotic cell cycle
import into cell
site of polarized growth
cell cortex
cell projection
mating projection
plasma membrane bounded cell projection
molecular adaptor activity
protein kinase activity
BP
CC
MF
p.adjust
0.001
0.002
0.003
Count
150
200
250
300
350
0.050 0.075 0.100 0.125 0.150
GeneRatio

I.

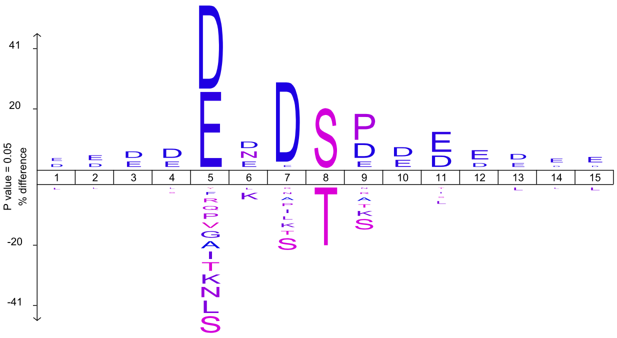
P value = 0.05
% difference
41
20
-20
-41
1 2 3 4 5 6 7 8 9 10 11 12 13 14 15

K.

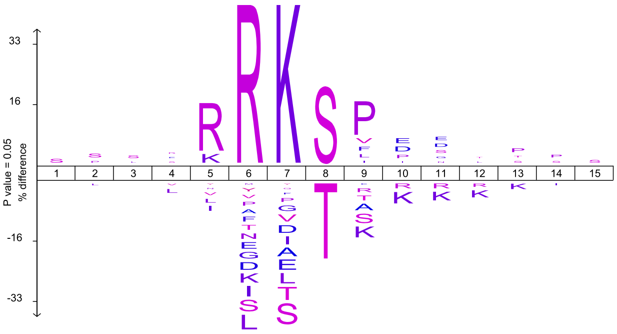
P value = 0.05
% difference
33
16
-16
-33
1 2 3 4 5 6 7 8 9 10 11 12 13 14 15

J.

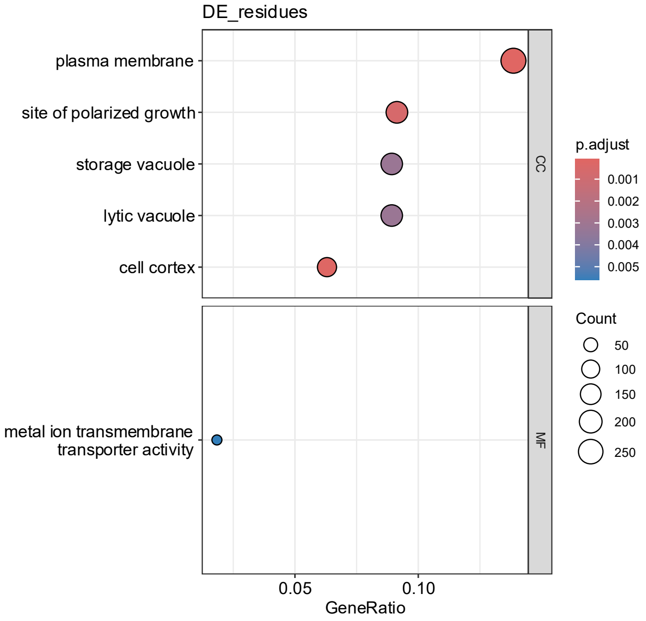
DE_residues
plasma membrane
site of polarized growth
storage vacuole
lytic vacuole
cell cortex
metal ion transmembrane transporter activity
CC
MF
p.adjust
0.001
0.002
0.003
0.004
0.005
Count
50
100
150
200
250
0.05
0.10
GeneRatio

L.

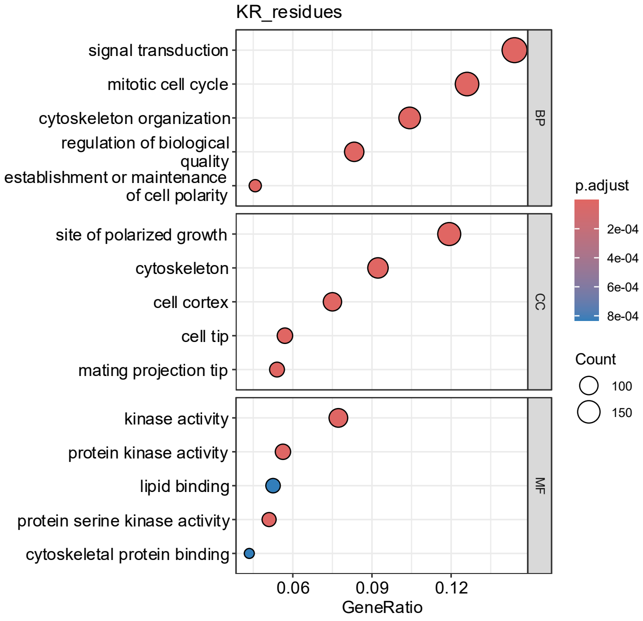
KR_residues
signal transduction
mitotic cell cycle
cytoskeleton organization
regulation of biological quality
establishment or maintenance of cell polarity
site of polarized growth
cytoskeleton
cell cortex
cell tip
mating projection tip
kinase activity
protein kinase activity
lipid binding
protein serine kinase activity
cytoskeletal protein binding
BP
CC
MF
p.adjust
2e-04
4e-04
6e-04
8e-04
Count
100
150
0.06
0.09
0.12
GeneRatio

M.

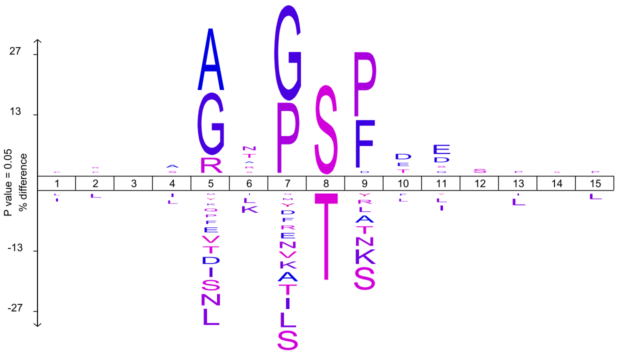

N.

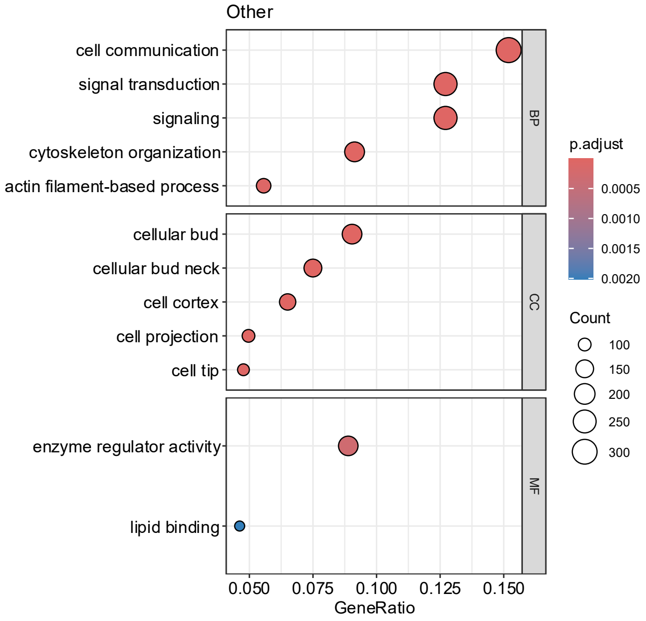

Figure S2. Motif and pathway enrichment analysis for all kinase motif classes. The iceLogos show the representation of amino acids at a given position either side of the central residue (serine or threonine) in the motif class (A, C, E, G, I, K and M).The dot plots display enriched pathways, as derived from clusterProfiler, in the group of proteins containing a motif from the given motif class (B, D, F, H, J, L and N).

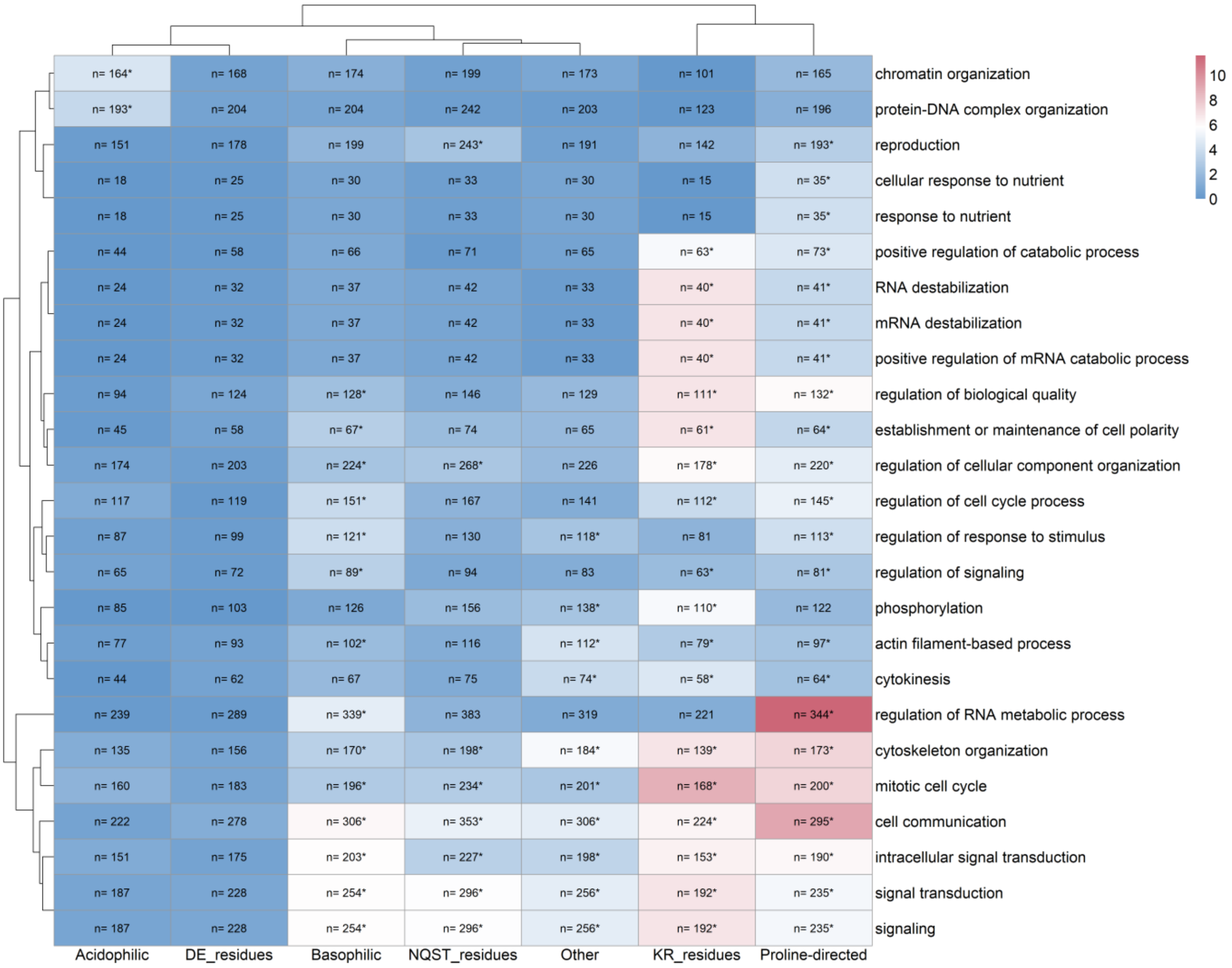

Figure S3. Heatmap of enriched gene ontology (GO) terms in the biological process category in the motif classes (up to the 'top 25' most significant terms). Gene counts and significance (* = p-value ≤ 0.01) for the GO term in each class are displayed.

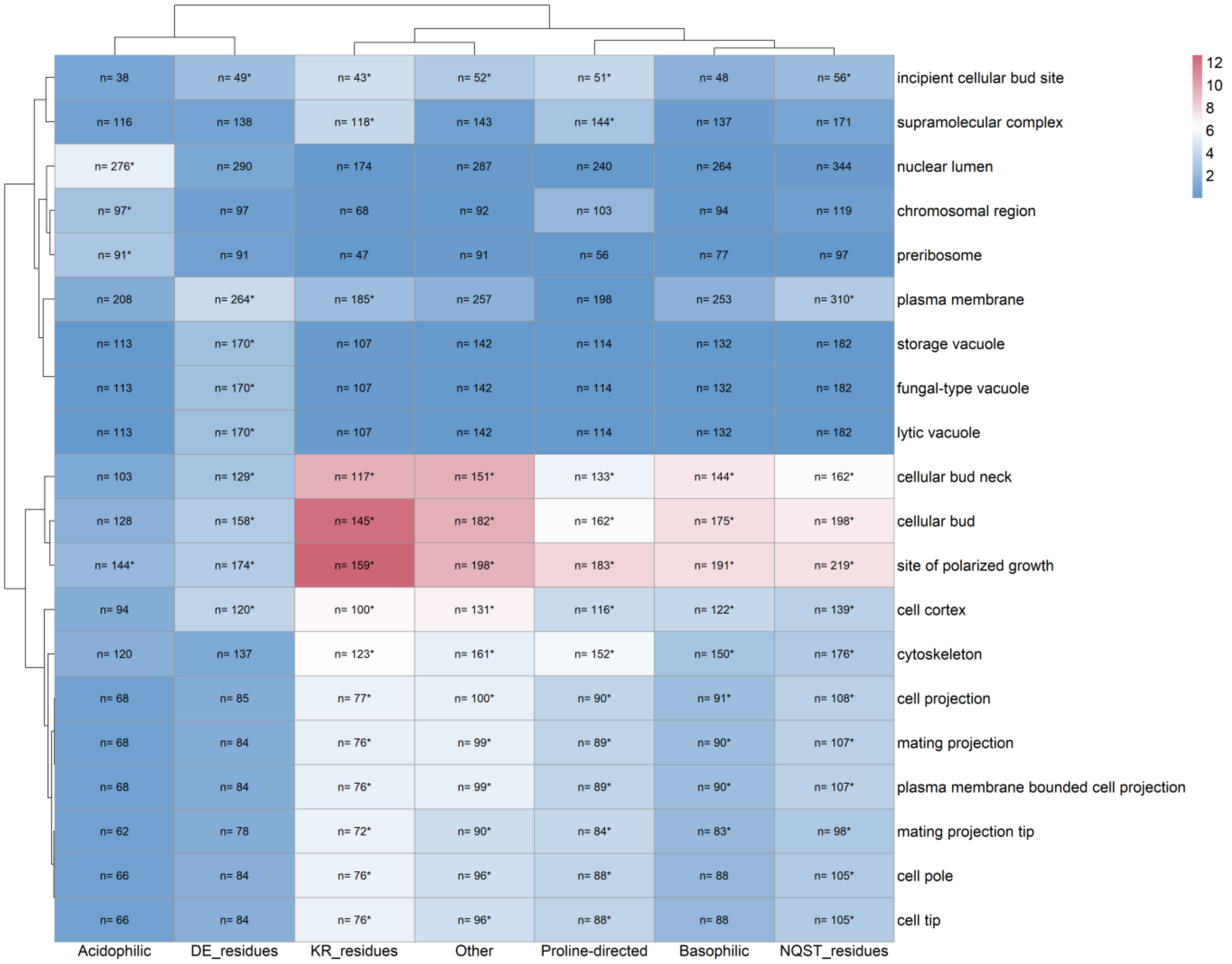

Figure S4. Heatmap of enriched GO terms in the cellular component category in the motif classes (up to the 'top 25' most significant terms). Gene counts and significance (* = p-value ≤ 0.01) for the GO term in each class are displayed.

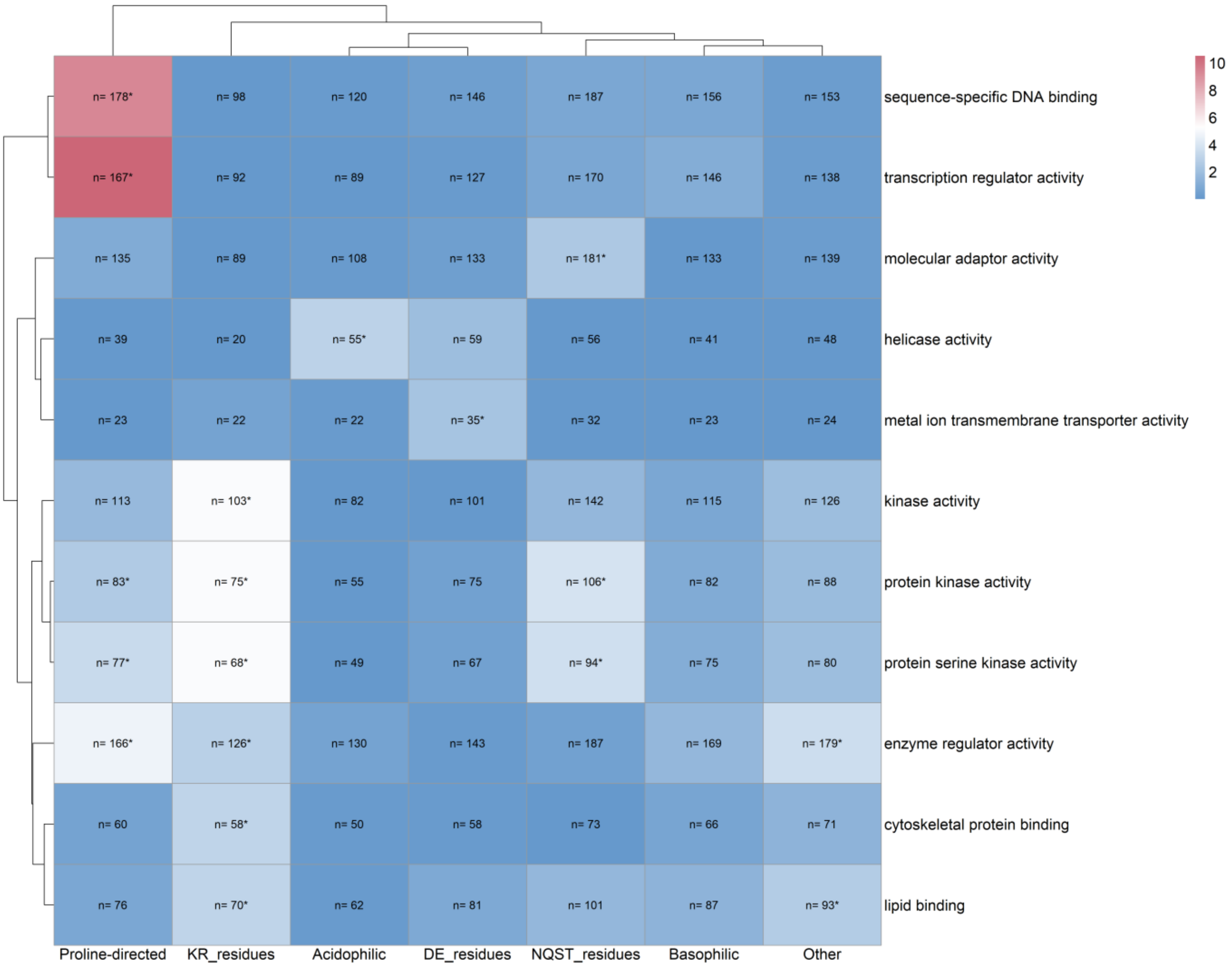

Figure S5. Heatmap of enriched GO terms in the molecular function category in motif classes (up to the 'top 25' most significant terms). Gene counts and significance (* = p-value ≤ 0.01) for the GO term in each class are displayed.

Disorder prediction analysis

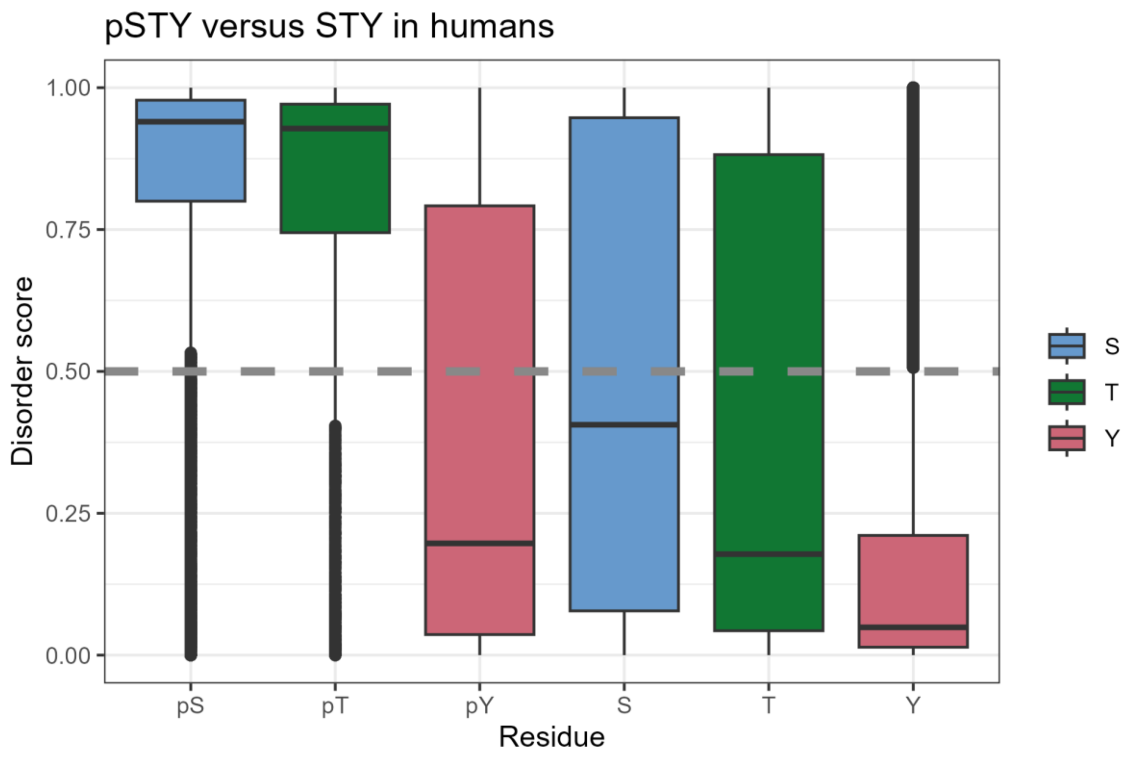

Figure S6. Disorder scores for gold standard pSTY sites compared to all STY sites in humans as analysed in Kalyuzhnyy et al., 2025 (45).

AlphaFold analysis

In Transposon Ty1-PL Gag-Pol polyprotein (Q12414) there are multiple phosphosites which overall contributed to many changes in the protein structure (Figure S7). Consequently, the overall structure of the protein changed from a disordered to an ordered conformation – this may perhaps represent a biologically plausible change in protein structure, although it should be noted that the polyprotein itself is only transiently produced, before protease activity creates separate Gag and Pol proteins.

In the case of Transposon Ty1-PL Gag-Pol polyprotein, the presence of helices in the phosphorylated state compared to the none phosphorylated state may be influenced by both the number of phosphorylated residues as well as the combination of nearby phosphorylated residues. Here, we modelled the first 50 residues of the Gag protein five times in each of the following states: phosphorylated at S3, phosphorylated at S7, phosphorylated at S3 and S7 and non-phosphorylated. In terms of the phosphorylated state models, the phosphosite(s) were encompassed in a helix in 5/5, 4/5 and 5/5 models respectively for the S3, S7, and S3 and S7 models. Conversely, in all five of the non-phosphorylation models, the protein was disordered near to the S3 and S7 residues (Figure S8).

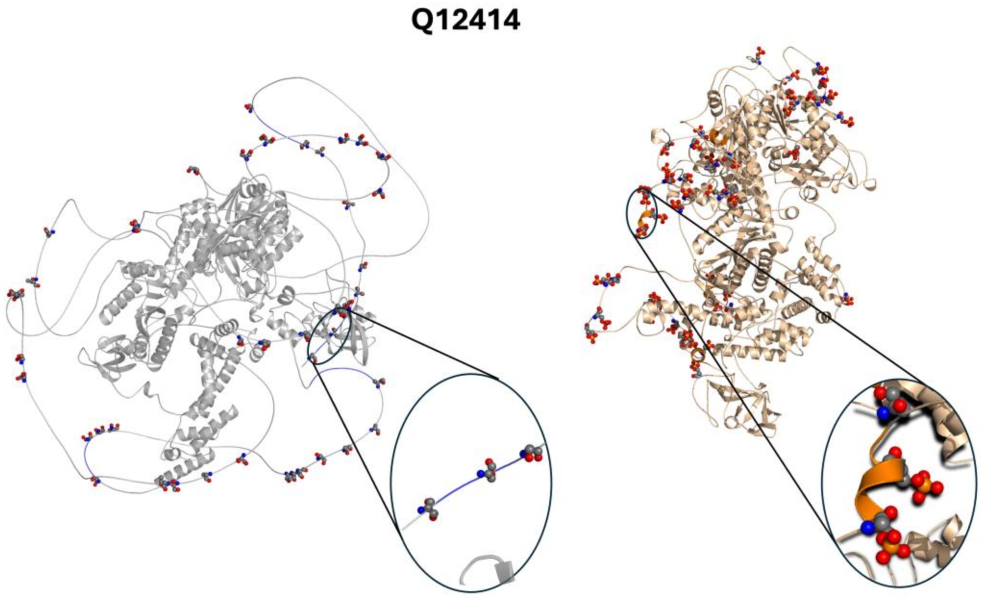

Figure S7. Transposon Ty1-PL Gag-Pol polyprotein (Q12414, phosphosites: residues 3, 7, 10, 13, 16, 28, 36, 38, 47, 177, 365, 371, 373, 375, 404, 405, 406, 409, 412, 416, 422, 480, 956, 960, 964, 965, 993, 995, 1005, 1006, 1032, 1051, 1055, 1059, 1081, 1085, 1093, 1095, 1099, 1105, 1140, 1162, 1181, 1187) is shown in its non-phosphorylated (left) and phosphorylated (right) states following structural alignment. Regions that undergo phosphorylation-associated conformational rearrangement are highlighted (residues 4-6, 8-9, 0, 39-45, 366-370 and 423-432), with the non-phosphorylated forms shown in blue and the phosphorylated forms shown in orange, while the surrounding structure is displayed as a pastel cartoon (grey for non-phosphorylated, wheat for phosphorylated). Phosphorylated residues are shown as sticks and spheres, coloured by atom type (C: grey, O: red, N: blue, P: orange).

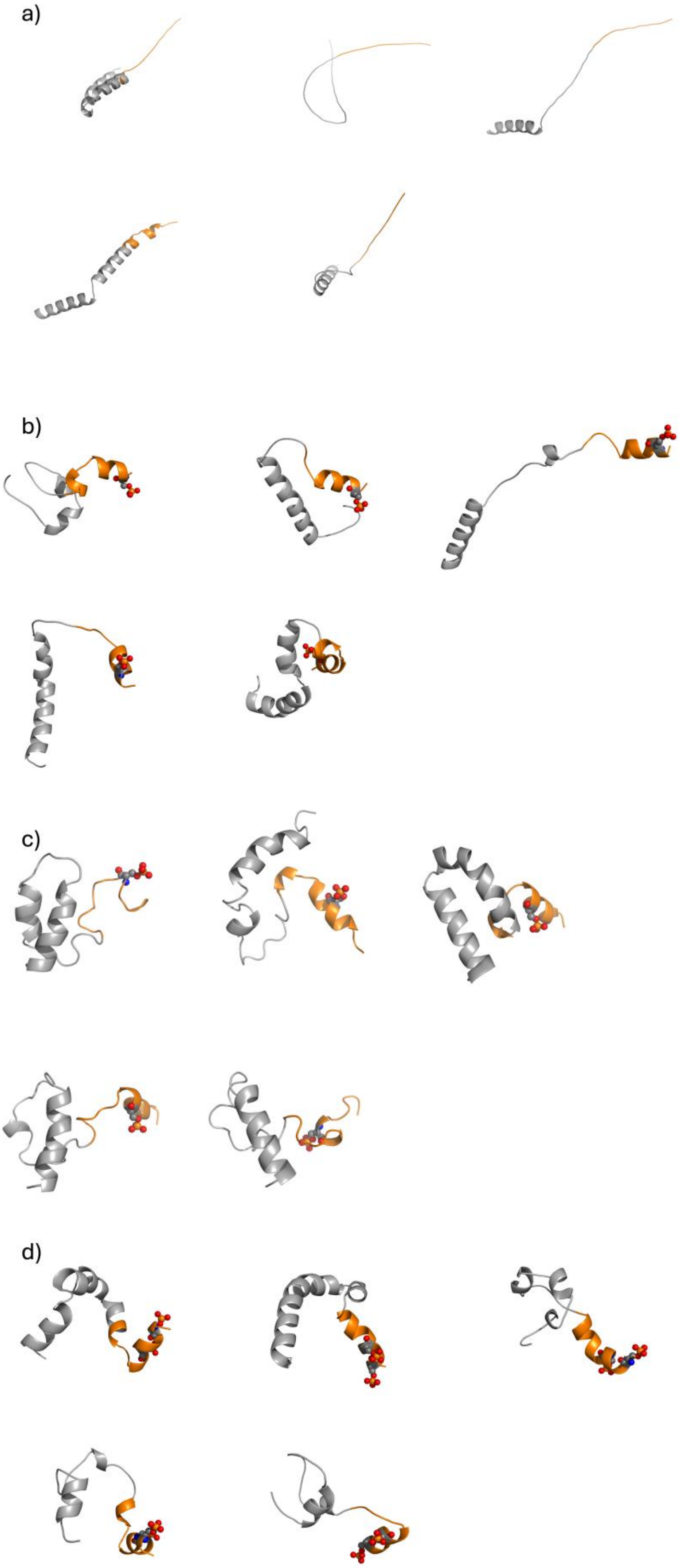
a)
b)
c)
d)

Figure S8. Structural Analysis of AlphaFold 3 predictions for the first 50 residues of Transposon Ty1-PL Gag-Pol polyprotein (Q12414) across variable phosphorylation states. a) Non-phosphorylated (No PTM), b) Ser3 phosphorylated (S3 only), c) Ser7 phosphorylated (S7 only), and d) Dual phosphorylated (S3+S7), were modelled using AlphaFold 3 (AF3). For each group, five independent AF3 runs were generated. The highest-ranking model was selected and aligned to the first run within that group to assess structural consistency and variability. All models are shown in cartoon representation. The local backbone is coloured light grey, while residues 1–15, containing the phosphorylation site(s), are highlighted in orange. Local PTM Site: For the PTM groups (S3, S7, S3+S7), the relevant serine residue(s) are explicitly rendered as sticks and spheres, with atoms coloured by type (Carbon: grey, Nitrogen: blue, Oxygen: red, Phosphorus: orange). For the No PTM group, the site is highlighted only by the orange local region colour, without stick/sphere representation.

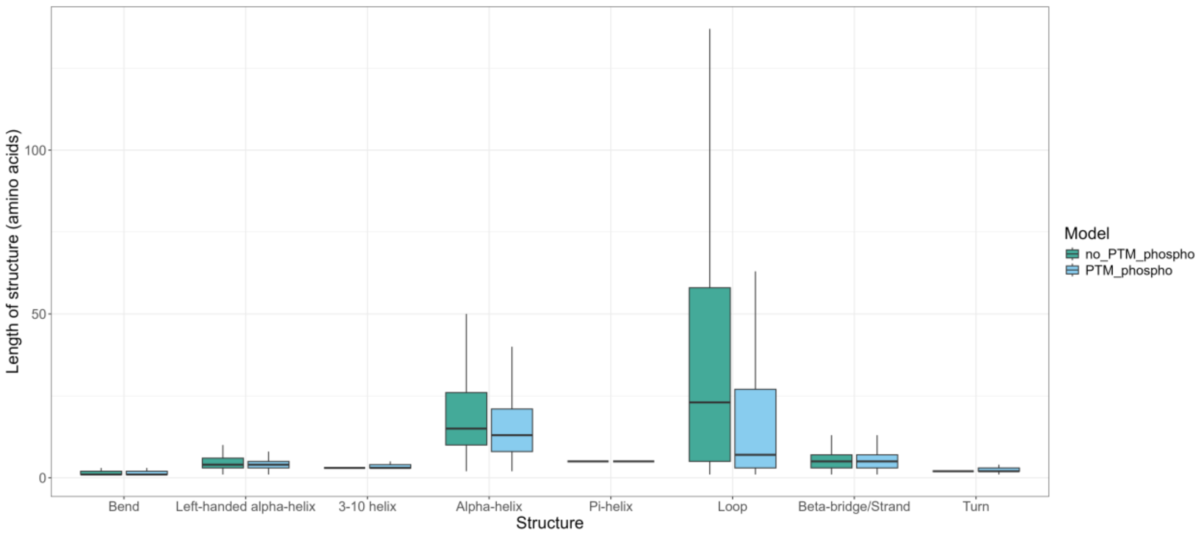

Figure S9. Length of secondary structure (amino acids) encompassing the phosphorylation site when the protein is modelled containing its 'Gold' phosphosites (model= PTM_phospho) compared to when the protein is modelled considering the amino acid sequence alone (model= no_PTM_phospho). Outliers have been excluded.

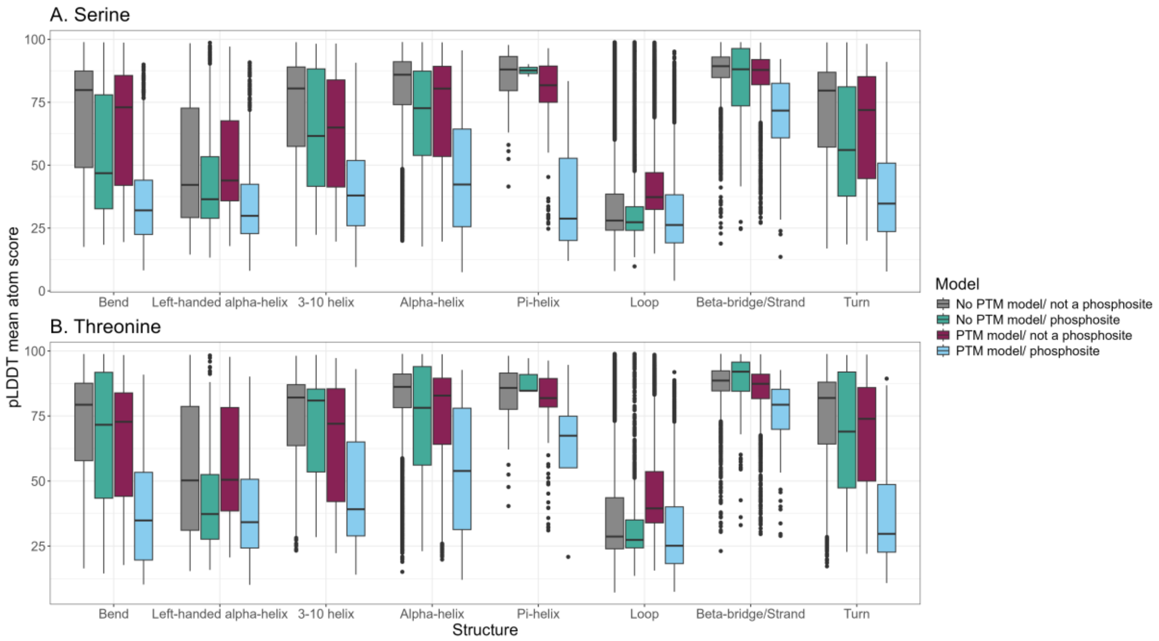

Figure S10. pLDDT score of serine and threonine residues at the secondary structure elements accounting for sites identified as 'Gold' phosphosites (green and blue), as well as

whether the protein has been modelled as the protein sequence alone (green and grey), or with the addition of the ‘Gold’ phosphosites (purple and blue).
